# Precisely controlling endogenous protein dosage in hPSCs and derivatives to model FOXG1 syndrome

**DOI:** 10.1038/s41467-019-08841-7

**Published:** 2019-02-25

**Authors:** Wenliang Zhu, Boya Zhang, Mengqi Li, Fan Mo, Tingwei Mi, Yihui Wu, Zhaoqian Teng, Qi Zhou, Wei Li, Baoyang Hu

**Affiliations:** 1 State Key Laboratory of Stem Cell and Reproductive Biology, Institute of Zoology, Chinese Academy of Sciences, Beijing, China; 2 University of Chinese Academy of Sciences, Beijing, China; 3 Institute for Stem cell and Regeneration, Chinese Academy of Sciences, Beijing, China

## Abstract

Dosage of key regulators impinge on developmental disorders such as FOXG1 syndrome. Since neither knock-out nor knock-down strategy assures flexible and precise protein abundance control, to study hypomorphic or haploinsufficiency expression remains challenging. We develop a system in human pluripotent stem cells (hPSCs) using CRISPR/Cas9 and SMASh technology, with which we can target endogenous proteins for precise dosage control in hPSCs and at multiple stages of neural differentiation. We also reveal FOXG1 dose-dependently affect the cellular constitution of human brain, with 60% mildly affect GABAergic interneuron development while 30% thresholds the production of MGE derived neurons. Abnormal interneuron differentiation accounts for various neurological defects such as epilepsy or seizures, which stimulates future innovative cures of FOXG1 syndrome. By means of its robustness and easiness, dosage-control of proteins in hPSCs and their derivatives will update the understanding and treatment of additional diseases caused by abnormal protein dosage.

## Abstract (web summary)

Altered dosage of developmental regulators such as transcription factors can result in disorders, such as FOXG1 syndrome. Here, the authors demonstrate the utility of SMASh technology for modulating protein dosage by modeling FOXG1 syndrome using human pluripotent stem cell-derived neurons and neural organoids.

## Introduction

Protein dosage fine tunes cell fate in development and engages in pathogenesis of certain diseases^1–3^. In human, modest alterations of protein abundance produce variable symptoms such as that in hypomorphic mutations or haploinsufficiency^4,5^. For a specified gene, half-loss, functional impairment or de novo gain of function either can affect protein dosage, which causes a broad spectrum of phenotypic manifestations^6–8^. Forkhead transcription factor 1 (*FOXG1*) in FOXG1 syndrome exemplifies such a scenario^9,10^. *FOXG1* is variably expressed at early stage of brain development^11^. In mice, while knock-out of FOXG1 causes preterm death and lack of ventral telencephalon^12^, haploinsufficiency only exhibits microcephaly with mild behavioral abnormalities^13,14^. In human, however, deletions or missense mutations on one allele of *FOXG1* cause severe neurodevelopmental disorders (FOXG1 syndrome)^15^. FOXG1 syndrome exhibits variable symptoms such as autism spectrum disorder (ASD), epilepsy, microcephaly (congenital or postnatal), severe intellectual disability, abnormal or involuntary movements, and unexplained episodes of crying^16–20^. Such diverse spectrum of neurological manifestations indicate that in patients of FOXG1 syndrome excitatory and inhibitory cortical neurons are variably constituted.

The dosage related and diverse outcomes of FOXG1 syndrome complicate the understanding of its pathogenesis. Because of difficulties in precisely dosage control of proteins using traditional knock-out and knock-down strategies, studying FOXG1 syndrome in rodents advances slowly. Differentiation of human pluripotent stem cells (hPSCs) can model early development, allowing for studying in a human context of development related disorders^21^. However, precise dosage control of a specific protein in hPSCs remains challenging. Lately, novel nuclease technologies such as clustered regularly interspaced short palindromic repeats/CRISPR-associated protein 9 (CRISPR/Cas9), advocate gene manipulation^22,23^. CRISPR nuclease (CRISPRn) induced monoallelic knock-out or point mutation can theoretically model haploinsufficiency in hPSCs^24,25^. However, both InDels and point mutations are based on the traditional DNA targeting methods, which may induce intrinsic compensation mechanism that disguises the direct consequences, or induce de novo phenotypes that further complicates the pathogenesis^26,27^. RNA targeting systems such as CRISPR interference or RNAi neither are suitable for precisely dosage control, because of the possibility of disproportional alterations of mRNA and protein^28,29^, let alone the labor-intensive selection of shRNAs or sgRNAs^30^. Thus, an inducible and tunable regulation system that acts exclusively at the protein level is favorable in hPSCs.

Protein abundance can be controlled through post-translational regulation using various chemical compounds^31–35^, such as that in small molecule-assisted shut-off (SMASh) technology. With a self-removing degron, SMASh effectively, reversibly, and precisely alters the abundance of proteins upon administration of small molecules to engineered cells such as HEK293 cells, rodent neurons or yeast^35^. SMASh system involves minimum genetic component and no fused proteins, which makes it preferable for genome editing. However, whether such a strategy works in hPSCs and can regulate endogenous protein for disease modeling remains unknown. In this study, we engineer hPSCs with SMASh tagged protein using CRISPR/Cas9 for precise dosage control, with which we can model protein dosage related disease such as FOXG1 syndrome.

## Results

### SMASh enables tunable shut-off of transgene in hPSCs

**Fig. 1:**
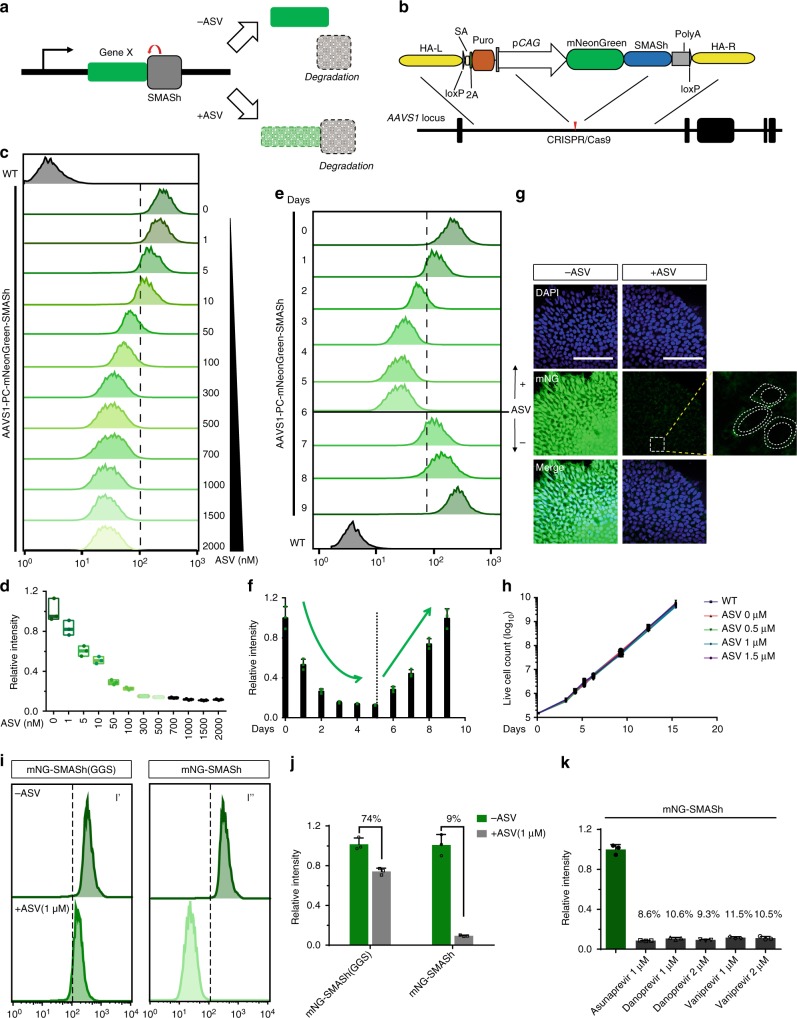
SMASh enables small molecule induced dose-dependent and tunable control of mNeonGreen in hPSCs. **a** Schematic outline of SMASh induced protein degradation. **b** Strategy for CRISPR/Cas9-mediated targeting to generate AAVS1-PC-mNeonGreen-SMASh hESC line. **c**, **d** Flow cytometry analysis (**c**) and quantification (**d**) (*n* = 3 biological replicates) of mNG intensity in AAVS1-PC-mNG-SMASh hESC line after 4 days of ASV treatment. Center line indicates the median; bounds of box indicate min to max. **e**, **f** Flow cytometry analysis (**e**) and quantification (**f**) (*n* = 3 biological replicates) of mNG intensity of AAVS1-PC-mNG-SMASh hESC line with ASV treatment. Dashed line indicates ASV withdraw. Cells were under 1 μM ASV treatment from day 1 to day 4 and withdraw from day 5. **g** Confocal images of AAVS1-PC-mNG-SMASh hESCs before and after treatment of ASV (1 μM) for 4 days. Scale bar, 100 μm. Right, zoom of dashed rectangle, dashed cycles depict 3 cells and nucleus. **h** Growth rate of AAVS1-PC-mNG-SMASh hESCs upon treatment with different concentration of ASV (0.5 μM, 1 μM, 1.5 μM, and 2 μM) for 3 passages (15 days) (*n* = 3 biological replicates). ASV does not affect the proliferation of hESCs. **i**, **j** Flow cytometry analysis (**i**) and quantification (**j**) (*n* = 3 biological replicates) of mNG intensity in AAVS1-PC-mNG-SMASh hESCs and AAVS1-PC-mNG-SMASh (GGS) after 4 days treatment of ASV (1 μM). **k** Quantification of mNG intensity in AAVS1-PC-mNG-SMASh hESCs after 4 days treatment of ASV as well as other two NS3 protease inhibitors, (*n* = 3 biological replicates). All error bars represent mean ± s.e.m. Source data are provided as a Source Data file

Small molecule-assisted shut-off (SMASh) is a technique in which proteins are fused to a self-removing degron that allows reversible and dose-dependent shut-off by administration of small molecules^35^. By default, SMASh self-cleaves and keeps the target protein from degradation. This process is instinct and can be blocked selectively and efficiently by the clinically available NS3 protease inhibitors such as Asunaprevir (ASV)^36^, Vaniprevir (VAV)^37^, and Danoprevir (DAV)^38^, resulting in the degradation of the fused protein (Fig. 1a). SMASh is a single-component system, which is suitable for genetic engineering. The nature of post-translational regulation also allows for rapid and tunable regulation of protein expression^35^.

To test whether SMASh works in hPSCs, we tagged mNeonGreen (mNG) with SMASh domain and inserted the construct into the “Safe Harbor” locus named AAVS1 using CRISPR/Cas9 (Fig. 1b). Upon treated with ASV, the fluorescence intensity is readily regulated by various concentrations of ASV (1~2000 nM) (Fig. 1c). SMASh is sensitive, and 1 nM of ASV can cause noticeable reduction; 500 nM induces maximum shut-off within 72 h (Fig. 1d). SMASh is also reversible, upon withdrawal of ASV (1 μM), mNG production was completely restored within 3–4 days (Fig. 1e, f). Residual fluorescence of mNG might be detectable (Fig. 1g) because it is optimized for stable expression^39^. Neither the insertion of SMASh domain nor administration of ASV affects the proliferation or pluripotency of the engineered hESCs, as is demonstrated in the nearly overlapping growth curves (Fig. 1h) and the expression of pluripotency markers such as OCT4, NANOG, and SOX2 (Supplementary Fig. 1c).

To confirm that mNG fluorescence intensity is precisely controlled by the SMASh tag, we replaced 41 residues in SMASh domain with “GGSGGGSGGGGS” sequence (refer as SMASh (GGS)) as reported^35^ (Supplementary Fig. 1a, b, d). As expected, upon administration of ASV, the shut-off efficiency of mNG-SMASh (GGS) is much lower, with 74% of fluorescence intensity remains, in contrast to the only 9% in mNG-SMASh hESCs (Fig. 1i, j and Supplementary Fig. 1d). Two other NS3 protease inhibitors (DAV, VAV), can also effectively shut down mNG expression (Fig. 1k and Supplementary Fig. 1e, f).

### Targeting endogenous gene in hPSCs with SMASh

**Fig. 2:**
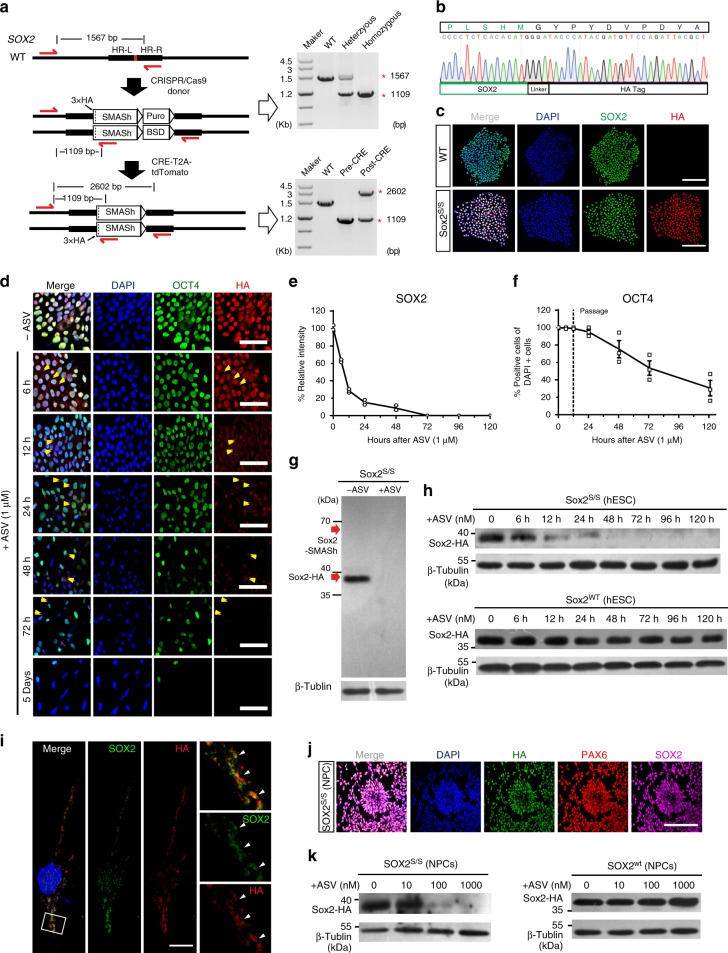
Tracking and dosage control of endogenous SOX2 in hPSCs. **a** Generation of *SOX2*^s/s^ hPSC lines using CRISPR and PCR genotype of a positive colony. **b** Sanger sequencing of a representative *SOX2*^s/s^ hPSC colony shows integration of HA-SMASh fragment. **c** Immunostaining for SOX2 (green) and HA (red) in H9-*SOX2*^s/s^ and WT H9 hESCs. Scale bar, 100 μm. **d**, **f** Immunostaining for OCT4 (green), HA (red) (**d**) and quantification (**f**) (*n* = 3 experimental replicates) of OCT4^+^ cells of H9-*SOX2*^s/s^ hESCs upon treatment with ASV (1 μM) showing degradation of endogenous SOX2 and declining of OCT4-expressing cells. Yellow arrow heads show the protein aggregates. Scale bar, 50 μm. Dashed line indicates the time of passage. **e**, **h** Western blot of H9-*SOX2*^s/s^ and WT H9 hESCs after ASV treatment for 5 days (**h**) and intensity analysis (**e**) (*n* = 3 experimental replicates) shows ASV (1 μM) shuts off of endogenous SOX2 in H9-*SOX2*^s/s^ hESCs. **g** Western blot of H9-*SOX2*^s/s^ hESCs showing efficient cleavage between SOX2 and SMASh without ASV and efficient degradation of SOX2 by SMASh upon administration of ASV (1 μM). In either case, no SOX2-SMASh fused protein exists. **i** Immunofluorescence images show aggregates of endogenous SOX2 protein after 48 h treatment of ASV (1 μM). White arrow heads indicate the co-localization of SOX2 (green) and HA (red). Right, zoom of the rectangle. Scale bar, 10 μm. **j** Immunostaining for PAX6 (red), SOX2 (magenta), HA (green) in H9-*SOX2*^s/s^ hESCs derived cortical neuronal progenitor cells (NPCs). Scale bar, 100 μm. **k** Western blotting showing that SOX2 protein level was dose dependently controlled by ASV in NPCs derived from H9-*SOX2*^s/s^ or wild type H9 hESCs. All error bars represent mean ± s.e.m. Source data are provided as a Source Data file

To test whether SMASh is effective for endogenous genes, we chose *SOX2* because it is involved in both pluripotency maintenance and neural differentiation, allowing us to study “induced dosage control” at various stages. We integrated the sequence of SMASh domain along with HA × 3 tag before the stop codon of endogenous *SOX2* using CRISPR/Cas9, so that the HA × 3 tag can indicate the endogenous SOX2 protein (Fig. 2a and Supplementary Fig. 2a). We developed a dual-antibiotic selection method, in which we offer two homologous donors with Puromycin or Blasticidin antibiotic marker, respectively in order to build homozygous SOX2-SMASh hESC lines (*SOX2*^s/s^) (Fig. 2a, b and Supplementary Fig. 2a). The targeting efficiency was up to 83.3% (35 of the 42 picked colonies are *SOX2*^s/s^) (Table 1 and Supplementary Fig. 2b). *SOX2*^s/s^ hESCs can express SOX2 protein with HA × 3 tag. Upon administration of ASV, SOX2 protein fades rapidly (Supplementary Fig. 2a). SMASh alone does not alter the pluripotency of *SOX2*^s/s^ hESCs and SOX2 can be easily detected by immunostaining with antibodies against either SOX2 or HA (Fig. 2c).

**Table 1:** Summary of targeting locus via CRISPR/Cas9

Target locus	Protein size (aa)	hPSC line	Donor	Colony number	Heterozygous	Homozygous	Targeting efficiency (%)	Homozygous efficiency (%)
AAVS1	236	H9	PC-mNG-SMASh	7	7		100	NT
SOX2	317	H9	SMASh-Puro SMASh-BSD	42	4	35	92.9	83.3
		H1	SMASh-Puro	21	10	8	85.7	38.1
		iPSC	SMASh-Puro	35	22	5	77.1	14.3
FOXG1	489	H9	SMASh-Puro	38	6	6	31.6	15.8
SOX17	414	H9	SMASh-Puro	39	1	2	7.70	5.13
ZNHIT1	154	H9	SMASh-Puro SMASh-BSD	36	13	15	77.8	41.7
TP53	393	H9	SMASh-Puro	41	18	7	61.0	17.1
B2M	119	H9	SMASh-Puro SMASh-BSD	32	6	21	87.5	65.6
AAVS1	1423	H9	PC-Cas9-SMASh	9	6		66.7	NT

SMASh is also effective in other pluripotent stem cell lines such as H1 and hiPSCs. These hPSC lines (H1-*SOX2*^s/s^, iPSC-*SOX2*^s/s^) exhibit normal growth, karyotypes, pluripotency, and ability of germ layer differentiation (Supplementary Fig. 2d, e, f and g).

Endogenous SOX2 was sensitively controlled by ASV in H9-*SOX2*^s/s^ hESCs (Fig. 2h). Upon treatment with 1 μM of ASV, SOX2 is declined rapidly in H9-*SOX2*^s/s^ hESCs within the first 48 h (Fig. 2d, e). Six hours after ASV administration, SOX2 tagged with SMASh appears in cytoplasm and forms aggregates (Fig. 2d). Forty-eight hours later, very few residual aggregate of SOX2 protein exists in nucleus (Fig. 2i). A similar change was also observed with western blotting (Fig. 2e, h). To exclude the possibility of inefficient separation of SOX2 and SMASh tag, we detected SOX2 and SOX2-SMASh fusion protein using HA antibody. We found that regardless of the administration of ASV, no fusion protein was detected (Fig. 2g), indicating an efficient cleavage between SOX2-HA protein and SMASh domain. We also observed a significant decline of OCT4^+^ cells after 24 h of ASV treatment, indicating exit of pluripotency accompanying SOX2 shut-off (Fig. 2d, f). We then differentiated *SOX2*^s/s^ hPSCs into neural progenitor cells (NPCs). *SOX2*^s/s^ NPCs express PAX6 as well as SOX2 (Fig. 2j). Upon administration of ASV (10, 100, and 1000 nM), SOX2 protein fades accordingly as shown with western blotting, indicating ASV sensitively and dose-dependently controls the abundance of SOX2 protein (Fig. 2k). These data suggest that SMASh can precisely regulate endogenous SOX2 protein in hPSCs and hPSC-derived NPCs.

**Fig. 3:**
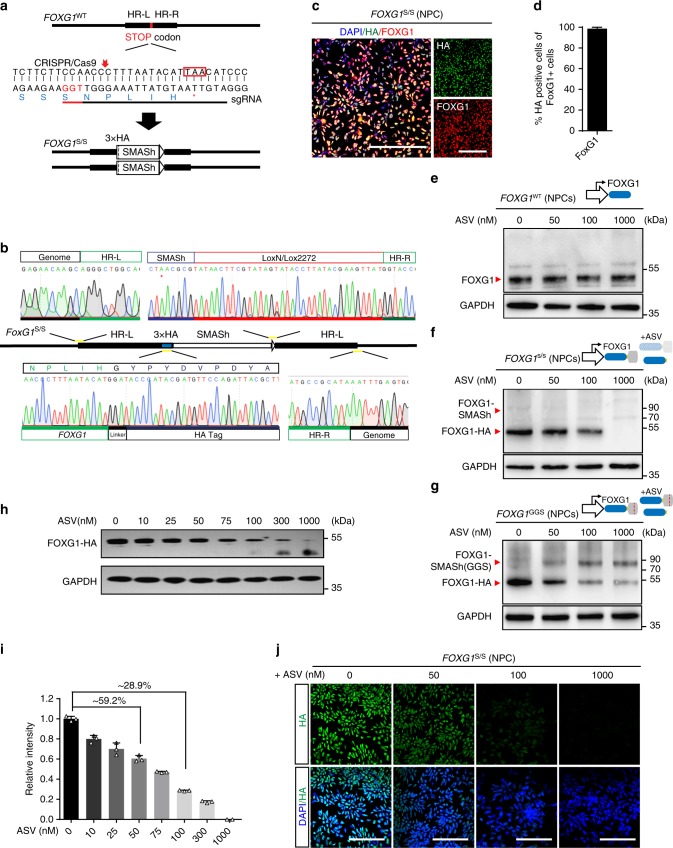
Generation and characterization of SMASh tagged FOXG1 hESCs. **a** Strategy for generation of *FOXG1*^s/s^ hPSC lines using CRISPR/Cas9. **b** Sanger sequencing of DNA fragment of a representative *FOXG1*^s/s^ hESC colony showing the integration of HA-SMASh. **c**, **d** Immunofluorescence images (**c**) and quantification (**d**) (*n* = 5 experimental replicates) of cortical neuronal progenitors derived from *FOXG1*^s/s^ hESCs for FOXG1 (red) and HA (green). **e**–**g** Western blotting for FOXG1 protein of WT hESC-derived NPCs (**e**), *FOXG1*^s/s^ hESC-derived NPCs (**f**) and FOXG1-GGS hESC-derived NPCs (**g**) under the treatment of ASV (0, 50, 100 , and 100 nM). **h**, **i** Western blot (**h**) and intensity analysis (**i**) (*n* = 3 experimental replicates) for *FOXG1* protein of *FOXG1*^s/s^ hESC-derived NPCs under treatment with different concentration of ASV (0, 10, 25, 50, 75, 100, 300, and 1000 nM). **j** Immunofluorescence images of FOXG1-expressing telencephalic progenitors for HA (green), DAPI (blue) upon treatment with different concentration of ASV (0, 50, 100, and 1000 nM), showing dosage regulation of FOXG1 protein. All error bars represent mean ± s.e.m. All scale bars 100 μm. Source data are provided as a Source Data file

To test the efficiency and robustness of SMASh with other proteins, we targeted a broad range of endogenous genes and transgenes such as transcription factor, SOX17 (Supplementary Fig. 3a and Supplementary Fig. 4a); zinc finger protein, ZNHIT1 (Supplementary Fig. 4d); oncogene, TP53 (Supplementary Fig. 4g); transmembrane protein, B2M (Supplementary Fig. 4j); transgene, spCas9, and FOXG1 (Fig. 3a, b), covering a wide range of protein sizes from 119 aa (ZNHIT1) to 1368 aa (spCas9) (Table 1). We engineered hPSCs with SMASh, targeting each gene using either dual-antibiotic selection method or traditional single antibiotic method. We found that dual-antibiotic selection method yield much higher efficiency of homozygous targeting (41.7–83.3% versus 5.14–38.1%) (Table 1). All SMASh tagged cell lines retain normal karyotype (Supplementary Fig. 4b, e, h, k). For each sgRNA, we sequenced the top 5 potentially off-targeting loci without off-targeting findings (Supplementary Fig. 4c, f, i, l). In definitive endoderm differentiated from the *SOX17*^s/s^ hPSCs, 1 μM of ASV nearly completely shut off SOX17 protein, verified by western blotting and immunostaining (Supplementary Fig. 3b, c). In other hPSC lines engineered with SMASh, the targeted protein with HA tag was readily seen by immunofluorescence and could be degraded after administration of 1 μM ASV (Supplementary Fig. 3d–k). In summary, SMASh can be adopted to hPSCs targeting multiple proteins, regardless of the nature, size or subcellular location of these proteins.

### SMASh fine-tunes the dosage of FOXG1 in hESC-derived NPCs

The sensitive dosage dependent shut-off of proteins by SMASh permits studying protein dosage and related developmental disorders, such as FOXG1 and FOXG1 syndrome. We thus proposed to investigate how reduced dosage of FOXG1 affects cortical GABA interneuron differentiation. We tagged FOXG1 with the SMASh or SMASh (GGS) domain and engineered hESC lines (refer as *FOXG1*^s/s^ hESCs and *FOXG1*^GGS^ hESCs) through genome editing (Fig. 3a, b). Upon differentiation into NPCs, both *FOXG1*^s/s^ NPCs and *FOXG1*^GGS^ NPCs express endogenous FOXG1 which can be labeled with HA without ASV (Fig. 3c, d and Supplementary Fig. 5b). For WT H9 hESC-derived NPCs, ASV cannot regulate endogenous FOXG1 protein (Fig. 3e), indicating ASV itself has no impact on FOXG1 expression in wild-type NPCs. For *FOXG1*^s/s^ NPCs, FOXG1-HA was dose-dependently regulated by ASV (Fig. 3f). Upon ASV treatment, FOXG1-SMASh fused protein was detected in *FOXG1*^GGS^ NPCs but not in *FOXG1*^s/s^ NPCs (Fig. 3f, g and Supplementary Fig. 5b), suggesting that mutant SMASh does not degrade FOXG1 protein properly. We further dissected the correlation of FOXG1 dosage and ASV concentration though western blotting. We revealed that SMASh can fine-tune FOXG1 abundance through the concentration of ASV. (Fig. 3h and Supplementary Fig. 5a). We chose 4 representative ASV concentrations (0, 50, 100, and 1000 nM) that induce different abundance of FOXG1 (100%, ~59.2%, ~28.9%, <1%, respectively) (Fig. 3i). We further confirmed this trend of alteration by immunofluorescence (Fig. 3j).

### FOXG1 regulates NPCs proliferation and MGE induction

**Fig. 4:**
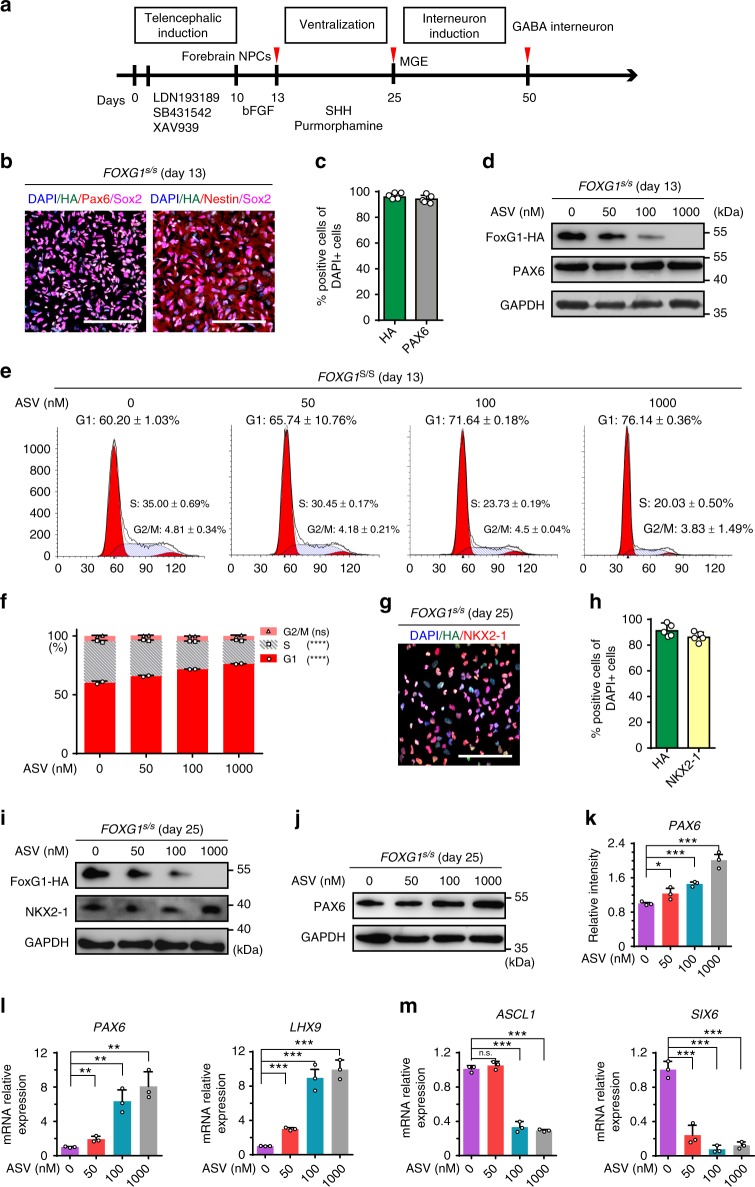
FOXG1 dose dependently regulates the proliferation of telencephalic progenitors and induction of cortical GABA interneuron. **a** Strategy for differentiation of cortical GABA interneurons from *FOXG1*^s/s^ hESCs. **b**, **c** Immunostaining (**b**) for telencephalic progenitor markers FOXG1-HA, NESTIN, PAX6, SOX2 and quantification (**c**) (*n* = 5 experimental replicates) for FOXG1-HA and PAX6 on day 13. **d** Western blotting showing in telencephalic progenitors FOXG1-HA reduced along with increasing of ASV concentration. PAX6 protein level remains the same. **e**, **f** Cell cycle analysis using FACS (**e**) and quantification (**f**) (*n* = 2 biological replicates) showing increase G1 and decreased S phase upon treatment with increased concentration of ASV (0, 50, 100, and 1000 nM). ****p < 0.0001 and n.s. not significant. Data were subjected to One-way ANOVA. **g**, **h** Immunostaining (**g**) and quantification (**h**) (*n* = 5 experimental replicates) for medial ganglionic eminence (MGE) progenitor makers FOXG1-HA (green), NKX2-1 (red) on day 25. **i, j** Western blotting to detect FOXG1-HA, NKX2-1 and PAX6 in MGE progenitors on day 25. **k** Intensity analysis for PAX6 protein on day 25 based on western blot (*n* = 3 biological replicates). *p < 0.05 and ***p < 0.001. Data were subjected to one-way ANOVA and Tukey’s multiple comparisons test. **l**, **m** qPCR analysis on day 25 (*n* = 3 biological replicates) under treatment of ASV (0, 50, 100, and 1000 nM) for dorsal prosencephalic marker *PAX6* (**l**), caudal forebrain marker *LHX9* (**m**), ventral telencephalic progenitor makers *ASCL1, SIX6*. **p < 0.01 and ***p < 0.001. Data were subjected to one-way ANOVA and Tukey’s multiple comparisons test. All scale bars 100 μm. Source data are provided as a Source Data file

We differentiated *FOXG1*^s/s^ hESCs into the telencephalic progenitors, medial ganglionic eminence (MGE) progenitors and finally cortical GABA interneurons as reported^40,41^ (Fig. 4a). On day 13 of differentiation, telencephalic progenitor markers such as *PAX6, FOXG1, NESTIN* and *SOX1* were extensively expressed (Fig. 4b, c). FOXG1 is dispensable for telencephalic induction as over 90% of the cells expressed PAX6 or SOX1 in all 4 groups (Supplementary Fig. 6a, c). Western results also showed that regardless of the FOXG1 dosage, PAX6 protein level remain unchanged (Fig. 4d). However, along with the decrease of FOXG1, cell cycle changes apparently with declined S phage and increased G1 phase accordingly (Fig. 4e, f), consistent with the microcephaly phenotype in FOXG1 syndrome patients, caused by impaired expansion of dorsal telencephalic progenitors^42^. In addition, FOXG1 insufficiency causes up-regulation of HES1, a downstream regulator of FOXG1 that is known to inhibit cell proliferation through Notch signaling, in a dose-dependent manner (Supplementary Fig. 6e)^43^. To draw a conclusion, in telencephalic progenitors, FOXG1 correlates with the proliferation by regulating cell cycle related genes such as HES1, in a continuous, dose-dependent manner.

Upon telencephalic progenitor induction, Sonic Hedgehog (Shh, 100 ng/ml) and purmorphamine (1 μM) induce MGE progenitors along dorsal-ventral (D-V) axes (Fig. 4a), during which the dorsal telencephalic marker PAX6 is down-regulated and ventral makers NKX2-1, FOXG1 are up-regulated^44,45^. On day 25, NKX2-1 and high-level of FOXG1 were induced (Fig. 4g, h). The reduced dosage of FOXG1 does not affect the generation of NKX2-1 expressing cells (Fig. 4i and Supplementary Fig. 6b, d), but causes PAX6 over-production (Fig. 4j, k). RT-PCR also showed that along with the reduction of FOXG1, *PAX6* and caudal forebrain marker *LHX9* mRNA level are increased (Fig. 4l), and that of ventral telencephalic progenitor markers such as *ASCL1, SIX6, DLX2*, and *OLIG2* are decreased (Fig. 4m and Supplementary Fig. 6g). All these results indicate that although SHH can successfully induce NKX2-1 expression in the hESC derived neural stem cells on day 25, reduced dosage of FOXG1 fails to initiate a downstream cascade for efficient ventralization, results in the over-production of non-ventral markers such as PAX6, LHX9 and the down-regulation of ventral transcription factors such as ASCL1, SIX6, DLX2, and OLIG2. It is noteworthy that the FOXG1 insufficiency only induce discrimination at ventralization stage, as on day 13 mRNA level of *DLX2* or *OLIG2* (Supplementary Fig. 6h) shows little difference.

### FOXG1 insufficiency blocks GABA interneuron induction

**Fig. 5:**
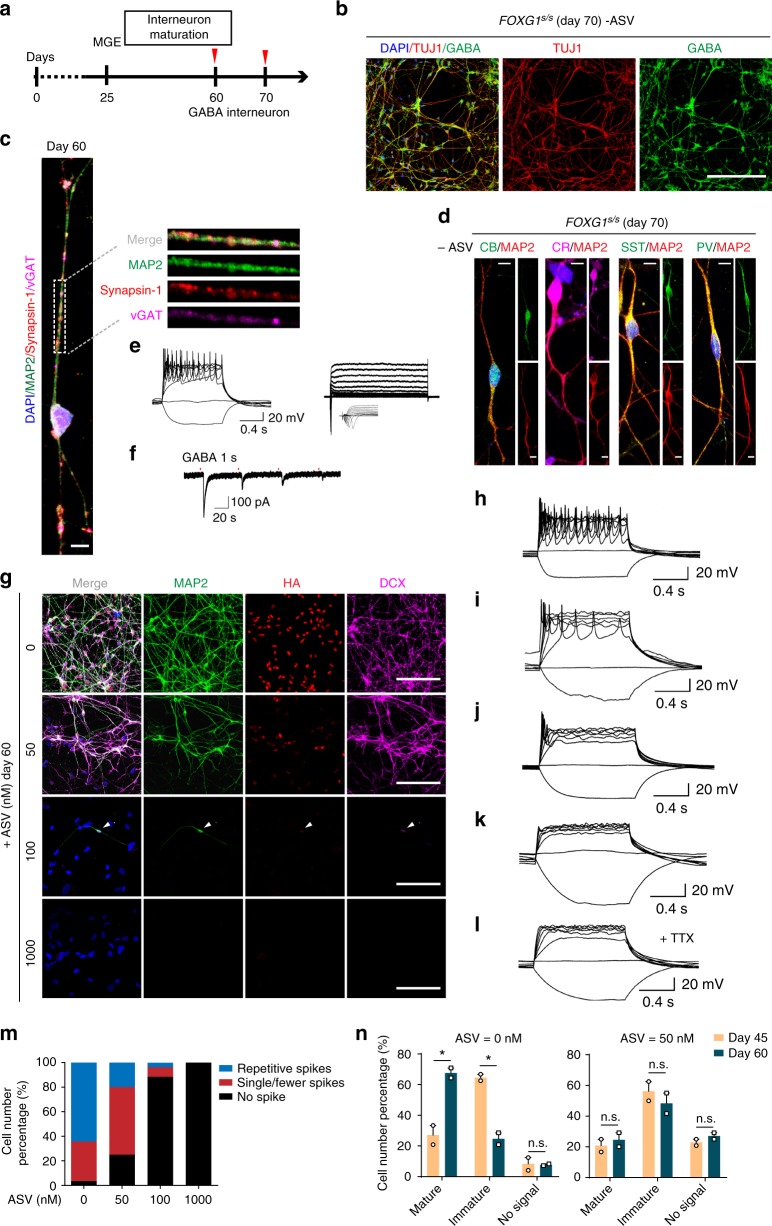
FOXG1 insufficiency impairs cortical GABA interneuron induction and maturation. **a** Schematic overview of induction and maturation of cortical GABA interneurons. Red arrow heads indicate the time that sample collected for analysis. **b** Immunofluorescence images on day 50 showing the expression of neuronal maker TUJ1 (red) and GABAergic inhibitory neuronal maker GABA (green). Scale bar, 100 μm. **c** Representative immunofluorescence image of a single neuron on day 60 showing the expression of mature GABAergic neuron makers MAP2 (green), vGAT (magenta), Synapsin 1(red). Right, zoom of dashed rectangle. Scale bar, 5 μm. **d** Representative immunofluorescence images of different subtypes of cortical inhibitory interneurons on day 70 of differentiation including Calbindin (CB), calretinin (CR), somatostatin (SST), parvalbumin (PV) neurons. Scale bar, 5 μm. **e** Representative AP firing patterns recorded on day 60 of a single neuron. **f** Whole-cell patch clamp reveals sIPSCs recorded from a single neuron on day 60. **g** Representative immunofluorescence images for MAP2 (green), FOXG1-HA (red), DCX (magenta) of GABA interneurons from each ASV treatment group (0, 50, 100, 1000 nM)) on day 60. Scale bar, 100 μm. **h**–**l** Representative AP firing patterns recorded on day 60 GABA interneurons from each ASV treating group (0, 50, 100, 1000 nM). **m** Quantification of the neuron maturity according recorded AP firing patterns of each ASV treating groups (0 nM, *n* = 28; 50 nM, *n* = 20; 100 nM, *n* = 26; 1000 nM, *n* = 6). **n** Quantification of the neuron maturity according recorded AP firing patterns of ASV = 0 nM group and ASV = 50 nM on day 45 and day 60. Results came from two independent experiments. Day 45: 0 nM, *n*_1_ = 24, *n*_2_ = 24; 50 nM, *n*_1_ = 24, *n*_2_ = 24. Day 60: 0 nM, *n*_1_ = 28, *n*_2_ = 24; 50 nM, *n*_1_ = 20, *n*_2_ = 24. *p < 0.05 and n.s. not significant. Data were subjected to an unpaired two-tailed *t* test. All error bars represent mean ± s.e.m. Source data are provided as a Source Data file

GABAergic interneuron induction started from day 25 (Fig. 5a). On day 60, TUJ1 together with GABA were expressed substantially in these cells (Fig. 5b), indicating efficient production of GABAergic neurons. For wild-type hESCs, we observed no difference of GABA or TUJ1 expression in GABA interneuron differentiation after long-term treatment of ASV (60 days) (Supplementary Fig. 7a). In mature cortical GABA interneurons, punctate presynaptic vGAT was expressed co-localized with GABA (Supplementary Fig. 7b) and together with other mature neuronal markers such as MAP2 and presynaptic marker synapsin 1 (SYP) (Fig. 5c). On day 70, the cells were differentiated into more distinct subtypes of cortical interneurons that express calbindin (CB), calretinin (CR), somatostatin (SST) and parvalbumin (PV) (Fig. 5d). We recorded the fire active potentials (AP) and spontaneous postsynaptic currents (sPSC) of a mature interneuron from day 60, indicating functional GABAergic-specific synapse formation (Fig. 5e, f).

In FOXG1 (ASV = 50) group, which yield ~59.2% of the abundance of FOXG1 protein (Fig. 3i), FOXG1 was expressed heterogeneously on day 60, and neuronal morphology remains unaltered (Fig. 5g and Supplementary Fig. 7d). Similar proportion of DCX^+^ and TUJ1^+^ cells were generated as that in the FOXG1 (ASV = 0) group (85.93 ± 7.53% versus 80.6 ± 8.0%) (Fig. 5g and Supplementary Fig. 7d, e). However, 100 and 1000 nM of ASV on FOXG1^+^ and TUJ1^+^ cells caused significant cell death, as well as obvious morphological alteration (Fig. 5g and Supplementary Fig. 7c-e), indicating that less than 30% of FOXG1 protein can neither sustain the identity of MGE cells nor generation of GABA interneurons.

To study whether FOXG1 dosage affect the maturity of GABA interneurons, we examined the electrophysiological properties of cells in each group using whole-cell patch recordings (ASV 0 nM, *n* = 28; 50 nM, *n* = 20; 100 nM, *n* = 26; 1000 nM, *n* = 6) (Fig. 5h–l). We found that in the FOXG1 (ASV = 0) group, 64.8% (*n* = 18) of the recorded mature AP firing properties with faster and consistent AP velocity, and 32.4% (*n* = 8) fire single or fewer spikes of action potentials, suggesting immature neurons. Only 3.57% (*n* = 1) of the recorded cells fail to show spikes of action potentials (Fig. 5m). Upon administration of ASV, the proportion of immature neurons and non-signal cells increased at 50 nM ASV, showing overall less mature feathers (Fig. 5m). Na^+^ currents also exhibit a similar pattern of alteration with the increased concentration of ASV induction (Supplementary Fig. 7f, g). Besides, to explore whether there is a developmental delay in FOXG1 (ASV = 50) group, we further recorded the AP firing patterns on day 45. In FOXG1 (ASV = 0) group, mature neurons increased significantly from day 45 to day 60, concomitant with the decrease of immature neurons, indicating the maturation of GABA interneuron. With 50 nM of ASV, on the other hand, a large number of cells without signal appeared and there was no tendency for neuron maturation (Fig. 5n). Our evidences indicate that the regulation of FOXG1 for GABA interneuron induction is more likely to be bi-phasic, as ~30% of FOXG1 dosage thresholds the production of MGE derived GABA interneurons, and ~60% of that produces some yet less mature TUJ1^+^ neurons.

### MGE organoids modeling ventral telencephalon development

**Fig. 6:**
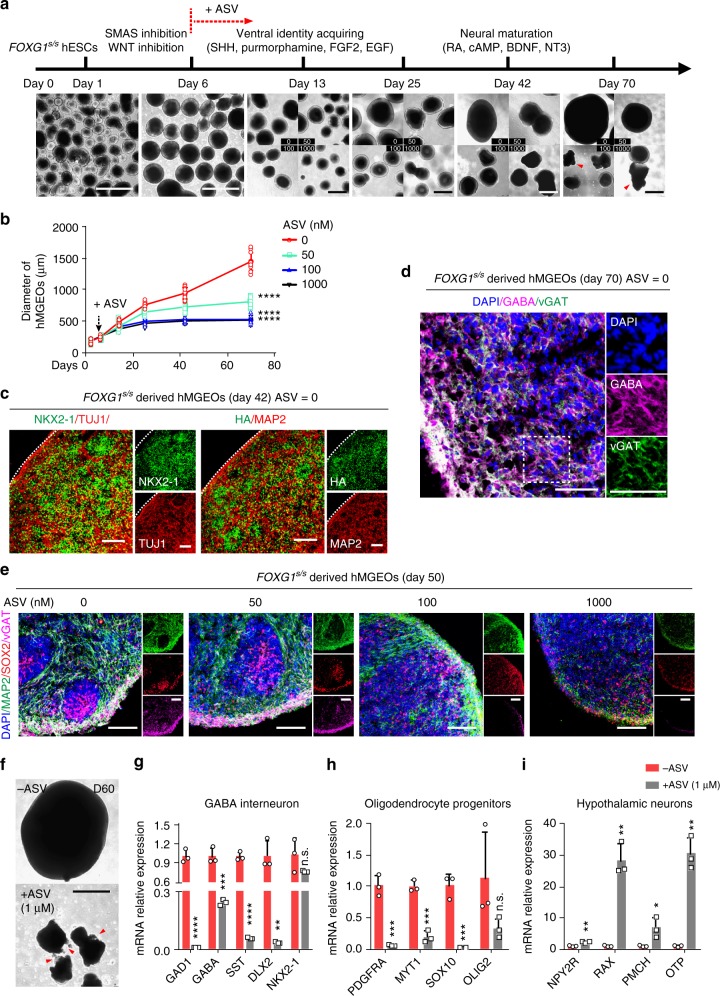
FOXG1 biphasically regulates GABA interneuron in hPSC-derived MGE organoids. **a** Schematic overview and bright field images of MGE organoids in different stages of differentiation. ASV treatment was started from day 6. Red arrow heads indicate shattered hMGEOs. Scale bar, 500 μm. **b** Quantification of the organoids’ sizes (*n* = 10 experimental replicates) from panel **a**. Dashed arrow indicates ASV treatment start time. ****p < 0.0001. Two-way ANOVA and Tukey’s multiple comparisons test. **c** Representative immunofluorescence images for NKX2-1 (left, green), TUJ1 (left, red), FOXG1-HA (right, green), MAP2 (right, red) of *FOXG1*^s/s^ derived hMGEOs on day 42, ASV = 0. Scale bar, 100 μm. **d** Representative immunofluorescence images for mature GABA interneuron markers vGAT (green), GABA (magenta) of *FOXG1*^s/s^ derived hMGEOs on day 70, ASV = 0. Right, zoom of dashed rectangle. Scale bar, 50 μm. **e** Representative immunofluorescence images for MAP2 (green), SOX2 (red), vGAT(magenta) of *FOXG1*^s/s^ derived hMGEOs from each ASV treatment group (0, 50, 100, and 1000 nM)) on day 50. Scale bar, 100 μm. **f** Representative bright field image of a hMGEO from ASV = 0 group and ASV = 1000 nM group on day 60. Red arrow heads indicate shattered hMGEOs. Scale bar, 500 μm. **g**–**i** qPCR analysis (*n* = 3 biological replicates) on the ASV = 0 and ASV = 1000 group at day 60 for GABA interneuron related genes (**g**), oligodendrocyte progenitor cells related genes (**h**) and hypothalamic neuron relate genes (**i**). *p < 0.05, **p < 0.01, ***p < 0.001, ****p < 0.0001 and n.s. not significant. Data were subjected to an unpaired two-tailed *t* test. All error bars represent mean ± s.e.m. Source data are provided as a Source Data file

We generated human MGE organoids (hMGEOs) to model ventral telencephalon development, which involves both mature GABA interneuron and MGE progenitors^46,47^. ASV was applied from day 6 to regulated FOXG1 protein during hMGEOs formation (Fig. 6a). Reduced dosage of FOXG1 severely hindered the expansion of neuronal progenitors, resulted in smaller hMGEOs in all ASV treatment groups (Fig. 6a, b). During hMGEOs formation, SHH and purmorphamine induce high levels of NKX2-1 and FOXG1-HA on day 40, together with neuronal makers such as TUJ1, MAP2 (Fig. 6c). At later stage (day 70), GABA and vGAT are expressed substantially in hMGEOs, suggesting maturation of GABA interneuron (Fig. 6d).

In ASV treatment groups, similar to that found in monolayer differentiation, GABA interneurons are induced as bi-phasic. At 100 and 1000 nM, ASV caused substantial decrease of TUJ1^+^ and vGAT^+^ cells (Fig. 6e). We observed massive cell death and significant organoids rupture after 50 days, together with increasing of Caspase 3^+^ cells (Supplementary Fig. 8a). In the survived organoids, we investigated what types of cells were born from NKX2-1 progenitors. During brain development, the NKX2-1 expressing MGE cells generates predominantly GABA interneurons^48^, as well as oligodendrocyte progenitor cells (OPCs)^49^. Besides, NKX2-1 expressing cells also generate hypothalamic neurons in ventral diencephalon^50^. We found in FOXG1 (ASV = 1000) group, mRNA of GABA interneuron related genes (*GAD1, GABA, SST,* and *DLX2*) and OPCs related genes (*PDGFRA, MYT1, SOX10,* and *OLIG2*) were substantially decreased (Fig. 6g, h), indicating differentiation deficit. Some hypothalamic neuron related genes (*NPY2R, RAX, PMCH,* and *OTP*), however, were up-regulated (Fig. 6i). These results suggest that the impaired induction of MGE progenitors results in large-scale cell death in late stage of GABA interneuron differentiation. Besides, FOXG1 deficiency turns the MGE cell fate to that of other brain regions, such as hypothalam.

**Fig. 7:**
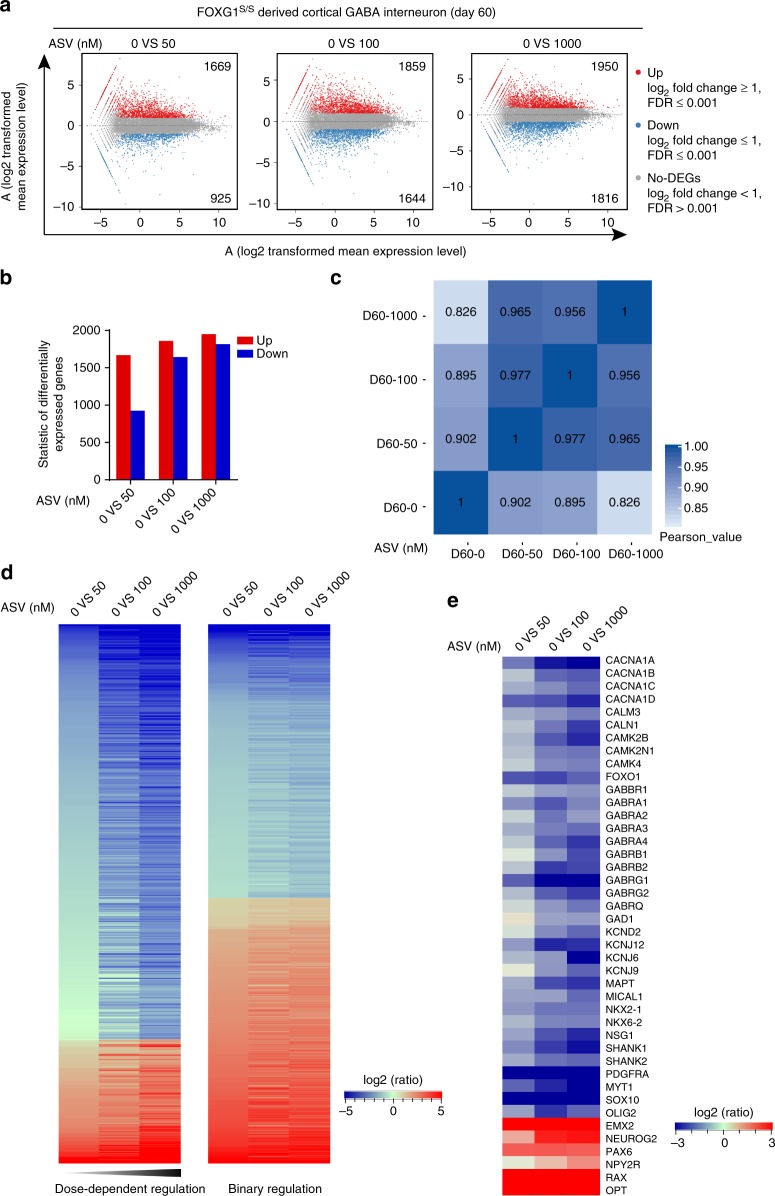
RNA-seq analysis of cortical GABA interneurons derived from hESCs with different FOXG1 dosage. **a** MA plots chart of differentially expressed genes (DEGs) compared to FOXG1 (ASV = 0) group. Red, blue and gray dots represent up-regulated, down-regulated DEGs and non-DEGs. **b** DEGs between each ASV treated and the untreated FOXG1 (ASV = 0) group. **c** Correlation heatmap showing weaker correlation between FOXG1 discriminated groups. **d** Expression profile presenting of two different clusters of DEGs. Left shows dose-dependent regulation by FOXG1, while right shows binary regulation by FOXG1. Coloring indicates the log2 transformed fold change. **e** Expression profile of key genes related to GABA neuron development and GABAergic synapses formation. Coloring indicates the log2 transformed fold change

RNA-Seq on GABA interneurons (day 60) indicate that compared to the FOXG1 (ASV = 0) group, the insufficiency of FOXG1 protein produced more than 1000 differentially expressed genes (DEGs) (Fig. 7a), and more DEGs in the group with less FOXG1 dosage (Fig. 7b). Heat map on Pearson Correlation indicate that groups with more discriminated FOXG1 exhibit much weaker correlation (Fig. 7c and Supplementary Fig. 8b). By analyzing the DEGs, we found that each group has a set of distinct DEGs (Supplementary Fig. 8c), suggesting each FOXG1 protein dosage results in distinct expression pattern during differentiation. The DEGs can be divided into two clusters (Supplementary Fig. 8d), with one showing dosage-dependent regulation by FOXG1 and the other typical bi-phasic (Fig. 7d). Key regulatory genes for GABA interneuron induction (*NKX2-1, NKX6-2, GAD1*, etc.) and functional GABAergic-specific synapse formation (*GABBR1, GABRA1, GABRB1, GABRG1, GABRQ, SHANK1* etc.) were down-regulated along with reduction of FOXG1, while genes of other brain region such as dorsal forebrain (*EMX2, NEUROG2, PAX6* etc.) and hypothalamic (*NPY2R, OTP, RAX* etc.) were up-regulated (Fig. 7e and Supplementary Fig. 8e).

### Modeling FOXG1 syndrome in hPSC-derived cortical organoids

Human cortical organoids (hCOs) produce various types of cortical neurons that mimic human organogenesis in vitro and can be used for disease modeling^51^. Although hCOs predominantly produce dorsal telencephalon cells (Supplementary Fig. 9g), a certain proportion of cells spontaneously become GABA interneurons^20,47^. We explored the dosage effect of FOXG1 on GABA interneuron induction in hCOs to model FOXG1 syndrome. Adverse effect of ASV was excluded because 1 μM ASV does not significantly affect organoid formation in wild-type hESCs as those organoids gradually gained similar sizes as that of control group from day 20 to day 70 (Supplementary Fig. 9a). RNA-Seq showed few DEGs in ASV treatment groups (Supplementary Fig. 9b, c). Radially organized VZ-like and SVZ-like structures were observed in hCOs on day 20 (Supplementary Fig. 9d, e). At later stage (day 70), iSVZ-like and oSVZ-like structures were further observed (Supplementary Fig. 9f), suggesting gradually maturation of hCOs.

**Fig. 8:**
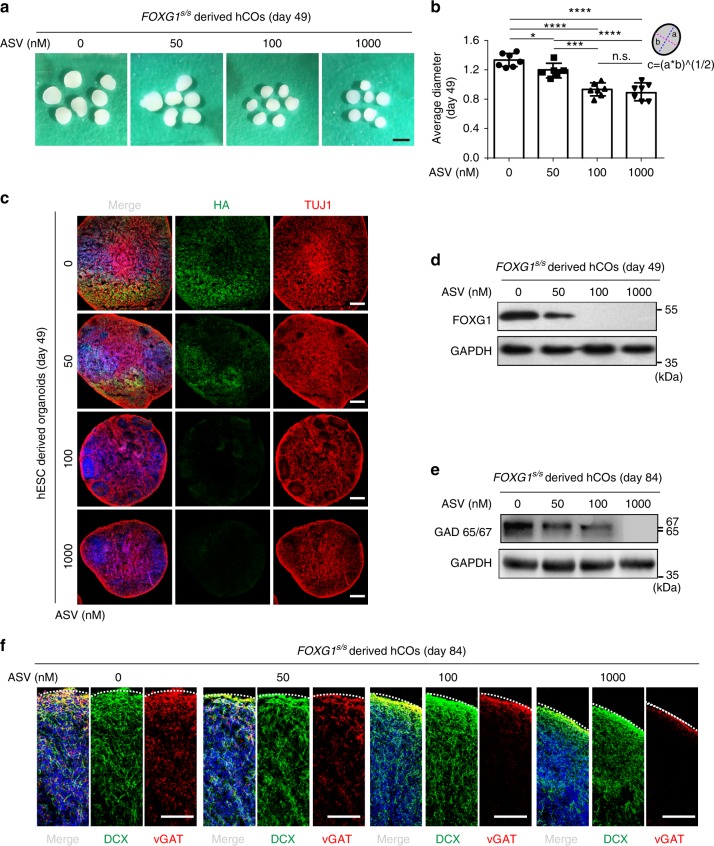
Model FOXG1 syndrome in hPSC-derived cortical organoids using SMASh. **a** Representative images of *FOXG1*^s/s^ hESCs derived hCOs on day 49 under the treatment of different concentration of ASV (0, 50, 100, and 1000 nM). Scale bar, 1 mm. **b** Quantification of the average diameter of the organoids on day 49. a, long semi-axis; b, short semi-axis. Average diameter *c* = 2*(a*b)^(1/2). Data are presented as dot plots of individual experiments. *p < 0.05, ***p < 0.001, ****p < 0.0001 and n.s. not significant. One-way ANOVA and Tukey’s multiple comparisons test. **c** Representative immunofluorescence images of organoids from each ASV treatment group (0, 50, 100, and 1000 nM) for HA (green), TUJ1 (red). Scale bar, 200 μm. **d** Western blot of *FOXG1*^s/s^ hESCs derived hCOs on day 49 to detect *FOXG1*-HA. **e** Western blot of *FOXG1*^s/s^ hESCs derived hCOs on day 84 to detect GAD65/67, showed decreasing of GAD65/67, as shutting off FOXG1 protein production. **f** Representative immunofluorescence for GABA interneuron maker vGAT (red) and neuronal maker DCX (green) in *FOXG1*^s/s^ hESCs derived hCOs on day 84. Scale bar, 100 μm. All error bars represent mean ± s.e.m. Source data are provided as a Source Data file

Upon administration of ASV, all four groups produced typical hCOs by the end of the 7th week, yet variable in sizes (Fig. 8a). Average diameter of hCOs was slightly smaller in FOXG1 (ASV = 50) group (*p* = 0.240) but significantly smaller in the other two groups (*p* < 0.0001) (Fig. 8b). These data are consistent with our findings in monolayer and hMEGOs differentiation (Figs. 4e, f and 6a, b), which might collectively well explain the microcephaly symptom in FOXG1 syndrome^52,53^.

FOXG1 protein level was sensitively regulated by ASV (Fig. 8c, d), indicating SMASh system works in hCOs. TUJ1 were widespread expressed in all 4 groups unlike directed differentiation of GABA interneuron, in which 30% FOXG1 dosage led to few TUJ1^+^ neurons (Fig. 5g), possibly because FOXG1 is dispensable to TUJ1 expression for some dorsal telencephalic neurons. To study how GABAergic interneuron were affected in hCOs, we detected the NKX2-1 expression on day 42. In FOXG1 (ASV = 0) group, a small proportion of NKX2-1^+^ cells exist (Supplementary Fig. 9h), while upon ASV administration, NKX2-1^+^ cells were further decreased (Supplementary Fig. 9h, i). At later stage (day 84) when mature GABAergic interneurons were developed, we detected the Glutamate Decarboxylase 65/67 (GAD65/67). GAD65/67 protein level reversely correlated to the concentration of ASV (Fig. 8e). Similarly, immunostaining on GABA presynaptic maker vGAT shows a similar pattern of bi-phasic regulation, whereas in the FOXG1 (ASV = 0) group vGAT^+^ GABAergic interneurons were extensively induced, the number of vGAT^+^ cells declined gradually as the ASV concentration is increased (Fig. 8f). Reduction of GABA interneuron mimics the cell population alteration in FOXG1 syndrome, which accounts for the presence of symptoms such as epileptic fits, seizure or ASD.

## Discussion

In order to study dosage related diseases such as FOXG1 syndrome, we adapt SMASh system for hPSCs via CRISPR mediated genome editing to precisely control the dosage of endogenous proteins. SMASh offers the first platform to precisely control the endogenous protein in hPSCs and to generate protein dosage insufficiency from mild reduction to fully knock out. To facilitate genome editing in hPSCs, we also develop a dual-antibiotic selection approach by which we can tag the SMASh domain to multiple coding genes efficiently. We prove that SMASh is specific, rapid and can be used to study the dosage effect of multiple genes at varies stages during hESC differentiation. Using SMASh, we reveal how FOXG1 dosage dependently affect the generation of GABAergic interneurons, which might explain the variable clinical manifestations of FOXG1 syndrome.

Controlling protein dosage via drug induced degradation or stabilization facilitates the study on protein function. However, previous strategies either potentially change the target protein structure with large regulatory domains, or require multiple components that difficult to be manipulated, are both unsuitable for genome editing in hPSCs^54–56^. For example, destabilizing domain (DD) technology can also control protein expression by a ligand in a dose-dependent manner^31^, but DD fuses with targeted protein permanently and shows limited small-molecule control over certain large proteins like Cas9 protein^57^. Recently developed “Trim Away” method for acute and rapid degradation of endogenous proteins requires no genetic modification^34^, but needs antibody electroporation which may be problematic when no suitable antibody is available, or for long-term induction such as during differentiation. SMASh has been proved efficient, fast and sensitive for a variety of applications^35^. It directly controls the protein degradation, which is superior to other methods including those targeting the transcription or translation^24,30,58^. The controllable self-cleavage of SMASh allows for minimal perturbation of target protein functions in hPSCs. Highly specific NS3 inhibitors without off-target concerns are safe in human, and minimally influence the hESCs as well^35^. In addition, we add a HA × 3 tag before SMASh domain, which allows for tracking of the targeted protein simply using HA antibody. This is particularly useful for studying genes lacking appropriate antibodies or proteins such as de novo genes in primates.

The diverse pathogenic variants in FOXG1 locus and variable dosages of its protein cause variable neurologic phenotypes in FOXG1 syndrome patients^16^. Using SMASh in hESC-derived organoids we successfully recapitulate features of FOXG1 syndrome, such as microcephaly and alterations of inhibitory interneurons leading to behavior disorders. We reveal how FOXG1 dose-dependently affects the proliferation of telencephalic progenitors and results to smaller telencephalon organoids. Our findings are in consistent with previous report that FOXG1 inactivation causes premature lengthening of telencephalic progenitor cell cycles^59^. FOXG1 abundance at around 60% causes mildly impaired MGE induction and delayed GABA interneuron maturation, and at 30% or less fails to induce cortical GABA interneuron in telencephalon organoids, which valuably complements to what has been found in FOXG1 knock-out mice^12,60^. FOXG1 insufficiency causes gradual deteriorating pathological phenotypes in MGE organoids during the differentiation of GABA interneuron. Abnormal MGE induction and insufficient GABAergic neurons differentiation explain certain behavioral abnormalities in FOXG1 syndrome patients such as the epileptic fits or seizures^53,61^. It is also possible to employ *FOXG1*^s/s^ hESCs as references to dissect more complicated pathogenesis, using iPSCs from patients exhibiting “gain of function” phenotypes owing to de novo mutations^16,62–64^.

SMASh is adaptable for broader applications. For example, based on SMASh tagged hPSC lines, other inducible systems such as Tet-On can be introduced into AAVS1 locus^65^ for dual small molecule orthogonal genetic manipulation. Such a system can be useful for bidirectional and temporal regulation. Also, heterozygous of SMASh tagged hPSCs can induce allele specific protein degradation, which is a promising tool to study imprinting genes^66^. It is also possible to apply this system to pharmaceutical purpose, for example screening drugs that can rescue the abnormal imprint gene expression.

## Methods

### Maintenance of hPSC cultures

H9, H1 human ES cells and human induced pluripotent stem cell line iPS (IMR90) were maintained in feeder-free culture condition on Marigel (Corning, 354277) coated 6-well plates in Essential 8 (E8) medium (Thermo Fisher Scientific, A1517001). Medium was changed daily and passaged every 5 days. Cells were regularly tested for mycoplasma.

### Construction of genome editing vectors

Mammal codon-optimized *Streptococcus pyogenes* wild-type Cas9 expressing plasmid was built base on pmaxGFP (LONZA). Cas9 sequence was obtained from PX458 (Addgene 48138)^67^. pCMV-intron-spCas9 (Supplementary Data 2) was constructed by replacing GFP in pmaxGFP with Cas9 cDNA. U6-sgRNA sequence was also obtained from PX458. pMini-sgRNA was built based on puc19 (Takara, 3219) using Gibson Assembly (NEB, E2611L). The pmini-sgRNA vector includes two BbsI restriction sites for rapid cloning of sgRNA. MIT CRISPR design tool (http://crispr.mit.edu) or DESKGEN Cloud (https://www.deskgen.com) was used to design the sgRNAs (Supplementary Data 2) and predict off-target sites.

In order to build SMASh donor vector containing SMASh domain, antibiotic selection makers and homologous recombination arms (HR arms), a master vector (SMASh-Puro-lox2272) was built first (Supplementary Data 2). The amino acid sequence of SMASh domain was obtained from previous article^35^. Human codon-optimized HA × 3-SMASh DNA sequence was synthesized by the Beijing Genomics Institute (BGI). PGK-Puro expression cassette was PCR amplified from OCT4–2A-eGFP-PGK-Puro (Addgene 31938)^68^. The “PGK-Puro” sequence was flanked by a pair of lox2272. All DNA fragments described above were constructed into a puc19 vector using Gibson Assembly. Then ~800 bp HR arms were PCR amplified from human genomic DNA and cloned into digested SMASh-Puro-lox2272 construct. For dual-antibiotic selection method, a second master donor with BSD resistance gene was constructed by replacing Puro and lox2272 in SMASh-Puro-lox2272 with BSD and loxN.

For the removal of the PGK-Puro cassette, pmax-CRE-T2A-tdTomato was built by replacing GFP in pmaxGFP with CRE-T2A-tdTomato. CRE cDNA was obtained from Cas9–2A-Cre (AAVS1 68468)^58^, a SV40NLS was added at the N-terminal to improve nuclear import efficiency and tdTomato was obtained from ptdTomato-N1 (Takara 632532). All plasmids were purified using EndoFree Plasmid Maxi Kit (QIAGEN 12362).

### Generation of SMASh tagged hPSC lines

To build SMASh tagged hPSC lines, we performed nucleofection using LONZA 4D-Nucleofector. hPSCs were cultured until reach 70~80% confluence. One hour before nucleofection, 10 μM ROCK-inhibitor Y27632 were added to the medium, then hPSCs were digested by Accutase (Thermo Fisher A1110501). After two times washes by PBS, 2 million cells were resuspended in 100 μl P3 reagent (Lonza V4XP-3024). 1.5 μg pmax-Cas9 plasmid, 1.5 μg pMini-sgRNA, 3 μg donor plasmid was used for a single nucleofection event and nucleofected by program CB-150. For dual-antibiotic selection method, 2.5 μg SMASh-Puro-lox2272 and 2.5 μg SMASh-BSD-loxN were used. It is worth noted that nucleofection efficiency can be various for different hPSC lines, so other programs should also be considered in order to maximize nucleofection efficiency. Beside CB-150, other programs like CA-137, CA-158, CM-113, CM119, CM-132 were also used for generation of different SMASh tagged hPSC lines, all of which could achieve 40–70% nucleofection efficiency.

After nucleofection, hPSCs were plated on Matrigel pre-coated 6-well plates in E8 medium. 1 μM Y27632 were used for the first 24 h. Puromycin (0.5 μg/ml) was added from day 2–10 until obvious colonies appeared. Two weeks after nucleofection, 48 colonies were picked for each cell lines for PCR genotype. For dual-antibiotic selection, puromycin selection was conducted from day 2–7 and blasticidin was added from day 5–14. For genotyping, cells were dissolved by QuickExtract™ DNA Extraction Solution (Epicentre, QE09050) and incubated at 65 °C for 10 min and then at 98 °C for 3 min to extract genome DNA. Genomic PCR was carried out using Q5^®^ High-Fidelity 2X Master Mix (New England Biolabs, M0492L). Finally, amplified DNA fragments of positive colonies were gel purified for Sanger sequencing.

For the removal of PGK-Puro or PGK-BSD cassette in identified positive colonies, cells were harvested and nuclofected as described above with 2 μg pmax-CRE-T2A-tdTomato plasmid. 48 h later, CRE protein expressing cells were enriched by FACS sorting (FACS-Aria, BD-Biosciences) followed by replating at a density of 300 cells/cm^2^ in Y27632 containing E8 medium. Individual colonies were picked and PCR genotyped as described above.

### Off-target analysis on SMASh tagged hPSC lines

For each sgRNA, we listed top5 potential off-target sites according MIT CRISPR design tool (http://crispr.mit.edu) or DESKGEN Cloud (https://www.deskgen.com). We designed PCR primers on the two flanks of each sgRNA target sequences for amplifying 200–400 bp DNA fragments. Then amplified fragments were purified and sequenced to identify whether there is any off-target mutation.

### Karyotyping analysis on SMASh tagged hPSC lines

SMASh tagged hPSC lines with 60~80% confluences were sent to the Chinese Academy of Medical Science & Peking Union Medical College in E8 medium for karyotyping analyzes and G-binding as a service. More than 20 metaphase spreads were analyzed.

### Teratoma formation and analysis

Cells were dissociated with Dispase (Thermo Fisher Scientific, 17105041) and harvested by centrifugation. Then cells were resuspended with ice-cold E8 medium/Matrigel (3:1) mixture at a density of 1 × 10^7^ cells/ml. Hundred microlitres of cell suspension was carefully injected into the muscle center in the hind leg of a 6- to 8-week-old SCID mice using a sterile glass needle under a sterile stereo microscope. After 8 weeks, mice were euthanized following the guidelines of the Institutional Animal Care and Use Committee of the Institute of Zoology, Chinese Academy of Sciences. Teratomas were removed from the mice, fixed, sliced, and stained with H&E for histology analysis. For each cell line, two teratomas were analyzed. SCID-Beige male mice purchased from Charles River Laboratories. All mice have free access to food and water and were housed in the institutional animal care facility (SPF) with a 12 h light-dark schedule. Data acquisition and analysis were performed by investigators blinded to experimental groups.

### Cortical GABA interneuron differentiation (monolayer)

hPSCs were cultured until reach ~80 confluence. Induction of neural progenitors was adapted from previous article^40^. On day 0, cells were dissociated with Dispase (1 U ml^−1^), then hPSCs aggregates were harvested by centrifugation and plated in ultra-low-attachment 10 cm dishes (Corning, 430591) to form embryoid bodies. From day 1, the telencephalic induction was started by changing the medium to NIM medium with dual SMAD inhibition consisting of DMEM-F12, 1× N2 supplement (A1370701), 1× B27 supplement, minus vitamin A (12587010), 1× GlutaMAX Supplement (35050061), 0.5X MEM Non-Essential Amino Acids Solution (11140050), 110 μM 2-mercaptoethanol (Sigma-Aldrich, M6250), 0.05% (v/v) Bovine Albumin Fraction V Solution (Sigma-Aldrich, B2064), 1x Penicillin-Streptomycin (10378016), 100 nM LDN193189 (Selleck, S2618), 10 μM SB431542 (Selleck, S1067), 2 μM XAV939 (Selleck, S1180). Media were replenished daily until day 8. Then, EBs were plated on Matrigel (BD, 356234) pre-coated 6-well plates and neuronal rosettes could be observed within a few days. From day 9, medium was changed to NPC medium consisting of DMEM-F12, 0.15% (w/v) Dextrose (Sigma-Aldrich, G6152), 110 μM 2-mercaptoethanol (Sigma-Aldrich, M6250), 1× N2 supplement, 1× B27 supplement without vitamin A, 20 ng/ml FGF2 (Peprotech). NPC medium was changed daily for 4 days until day 13.

On day 13, neuronal rosettes were picked mechanically and dissociated with Accutase to single cells and replated onto Matrigel pre-coated 6-well plates at a density of 3.0 × 10^5^ cells per cm^−2^ in NPC medium. On the next day, medium was changed to the ventral patterning medium consisting of DMEM-F12, 0.15% (w/v) Dextrose, 110 μM 2-mercaptoethanol, 1x N2 supplement, 1x B27 supplement without vitamin A, 1× Penicillin-Streptomycin, 100 ng/ml recombinant SHH (PeproTech, 100–45), and 1 μM purmorphamine (Selleck, S3042). Medium was replenished every other day. On day 25, MGE-like neuronal progenitor cells were dissociated with Accutase and pipette into clumps of 5–50 cells and plated on Matrigel pre-coated 12-well plates. The medium was changed to neuronal differentiation medium (NDM), containing 50% DMEM-F12 medium and 50% Neurobasal medium (21103049), supplemented with 0.5× N2 supplement, 1× B27 supplement (17504044), 1× Glutamx, 0.5× MEM-NEAA, 0.025% (v/v) human insulin solution (Roche, 11376497001), 55 μM 2-mercaptoethanol, 1× Penicillin-Streptomycin, 0.2 mM Adenosine 3′ 5′-cyclic monophosphate (Sigma Aldrich, D0260), 200 mM ascorbic acid, 20 ng/ml NT-3 (PeproTech, 450–03), 20 ng/ml BDNF (PeproTech, 450–02), 20 ng/ml GDNF (PeproTech, 450–10). Medium was replenished every 3 or 4 days.

### Generation of human MGE organoids (hMGEOs)

hMGEOs differentiation was adapted from previous reports^46,47^. Briefly, hPSCs were processed the same as monolayer differentiation protocol described above for the first 6 days. Form day 7, organoids were transferred to a Sunflower Mini-Shaker (Grant-Bio) and medium was changed to the ventral patterning medium with 20 ng/ml FGF2 and 20 ng/ml EGF. Medium was replenished every other day. On day 25, the medium was changed to neuronal differentiation medium (NDM), medium was replenished every 3 or 4 days.

### Generation of hCOs

Cortical organoids were generated from hPSC lines using a reported protocol^69^, with some modifications. Briefly, hPSCs were dissociated to single cells by exposed to Accutase for 5 mins at 37 ℃. Collect cells and plate 8000 cells in each well of PrimeSurface 96 V Plate (Sumitomo Bakelite, MS-9096VZ) to form equal prime EBs, followed by careful centrifuge at 300 × *g* for 3 min. Wells contained neuronal induction medium containing 50% NIM (DMEM/F12, 1× N2 supplement, 1× NEAA, 1× GlutaMAX, Heparin (Sigma, 2ug/ml)) and 50% Essential 8 medium with dorsomorphin (compound C; Merck, 5 µM), SB-431542 (10 μM) and Y-27632 (10 μM) for first six days. The medium was changed on day 7 with neural medium, containing Neurobasal, 1× B-27 supplement and 1× GlutaMAX, which were supplemented with 10 ng/ml FGF-basic and 20 ng/ml EGF. To promote growth and differentiation, organoids were transferred into ultra-low-attachment 10 cm dishes on day 8 using a cut 1000 μl pipette tip (Nunc). These organoids were cultured using a Sunflower Mini-Shaker (Grant-Bio) with every other day medium change (day 8 to day 22). To promote neural progenitors differentiation, organoids subsequently cultured in neural medium was supplemented with 10 ng/ml BDNF, 10 ng/ml GDNF, 10 ng/ml IGF-1 and 20 ng/ml NT3 starting at day 23. Medium changes every 3 or 4 days.

### Definitive endoderm differentiation

Differentiation was started when hPSCs reach 40–60% confluence. On day 1, E8 medium was changed to DE induction medium consisting of RPMI 1640 Medium (Thermo Fisher Scientific, 61870044), 1× MEM Non-Essential Amino Acids Solution, 1× Penicillin-Streptomycin, 100 ng/ml Activin A (PeproTech, 120–14), 3 μM CHIR99021 (Selleck, S2924). This medium was changed daily until day 3. From day 4–6, medium was changed to DE expansion medium consisting of RPMI 1640 Medium, 1× MEM Non-Essential Amino Acids Solution, 1× Penicillin-Streptomycin, 50 ng/ml Activin A. Cells had shown the typical morphology of definitive endoderm cells.

### Immunofluorescence staining

Coverslip cultures were fixed in 4% (w/v) paraformaldehyde (Affymetrix) at room temperature (RT) in 1× PBS for 15 mins at room temperature (RT), washed with washing buffer (1× PBS, 0.3% Triton-100) for three times (5 min for each time) at RT. Then cultures were incubated for 1 h in blocking buffer (1× PBS, 10% donkey serum, 0.3% Triton X-100). Then the samples were incubated at 4 °C overnight with primary antibodies, diluted in 5% donkey serum and 0.1% (v/v) Triton X-100 in PBS. The next day, cells were washed three times in 1× PBS for 5 mins each. The samples were subsequently incubated with secondary antibodies at room temperature, diluted in 5% donkey serum in PBS for 1–2 h. After three times PBS washes, Nuclei were counterstained with DAPI (4′, 6-diamidino-2-phenylindole) at room temperature for 10 min and mounted on glass slides.

Organoids were collected in 1.5 ml Eppendorf tubes, fixed in 4% paraformaldehyde (PFA) at RT for 30 min, washed three times with PBS (5 min incubation at RT for each wash). Then samples were transferred to 30% sucrose solution for incubation overnight at 4 °C. On the next day, sucrose solution was removed and organoids were equilibrated with O.C.T compound at RT for 15 mins. Next, organoids were placed in tissue base molds and embedded within O.C.T compound at −20 °C. Organoids blocks were stored at −80 °C or used for cryosectioning to obtain 20 μm slices using freezing microtome. Cryosection was washed with washing buffer (1× PBS, 0.3% Triton-100) for three times (5 min for each time) at RT. The following procedure is the same as described above for coverslip cultures. Images were taken under a Carl Zeiss LSM710 confocal microscope and processed using ZEN 2012 software. Cell were manually counted using ImageJ. For each value, at least three images were taken for analysis for every experimental condition. Antibodies, catalog numbers and dilutions are presented in Supplementary Data 1.

### Flow cytometry

Cells were dissociated with Accutase and further dispersed into single cells. Then washed three times with cell staining buffer (BioLegend, 420201). For B2M^S/S^ hESCs, cells were fixed in 4% (v/v) paraformaldehyde in PBS for 20 min. After three times of washing, B2M staining (FITC anti-human β2-microglobulin Antibody, BioLegend, 316304) were performed for 60 min on ice in FACS buffer. 30,000 events were captured on BD FACS AriaIII flow cytometer, and data were analyzed with FlowJo_V10. For detect and measure median fluorescence intensity (MFI) of AAVS1-PC-mNG-SMASh hESCs or AAVS1-PC-mNG (GGS)-SMASh hESCs, FACS were performed on live cells without fixation. For the quantification of relative mNG intensity, mNG positive cells were gated and the MFI was measured using FlowJo software. Intensity analysis was performed on three independent experiments. Graphs were made by GraphPad Prism 6. The FACS sequential gating strategies are indicated in Supplementary Fig. 12.

To analyze the cell cycle of a cell population using PI staining, single cells were harvested as described above. Cells were collected in 1.5 ml Eppendorf tubes and then resuspended in 1 ml of chilled 70% ethanol overnight at −20 °C. The next day, cells were washed and resuspended in 200 μl Muse cycle reagent (Muse cell cycle kit, Millipore, MCH100106) for 30 min at RT in the dark. Finally, cell cycle profiles were measured using a BD FACSCalibur flow cytometer. The percentage of cells in each cell cycle phase was came from three independent experiments and determined using ModFitLT V3.2.

### Western blotting

Samples were lysed using by RIPA Lysis and Extraction buffer (Thermo Fisher Scientific, 89901) containing protease inhibitor (Roche, 05892791001). Protein was quantified and 20 μg of each lysate were loaded per lane of a NuPAGE™ 4–12% Bis-Tris Protein Gel (Thermo Fisher Scientific, NP0335PK2). Samples were separated on 200 V for 35 min. Protein were then transferred to Nitrocellulose Blotting Membranes (PALL, P66485) at 200 mA for 2 h. The membranes were blocked in 5% evaporated milk in Tris-based saline with Tween 20 (0.05% TBST) buffer for 1 h and followed by incubating with primary antibodies overnight at 4 °C. The next day, membranes were washed three times with TBST (5 min each time) and incubated with HRP conjugated secondary antibodies for 2 h at RT. Protein bands were visualized using SuperSignal West Pico PLUS Chemiluminescent Substrate (Thermo Fisher Scientific, 34580) and blot image were captured by Tanon 5200 Automatic chemiluminescence image analysis system. Quantification of band intensities of FOXG1 or SOX2 was performed using imageJ on 3 independent experiments. The uncropped versions of western blots presented in the figures can be found in Supplementary Fig. 10 and Supplementary Fig. 11. Antibodies, catalog numbers and dilutions are presented in Supplementary Data 1.

### RNA isolation and qRT-PCR

Total RNA was extracted from approximately 10^6^ cells using the miRNeasy Mini Kit (QIAGEN, 217004). Then first-strand cDNA was produced using PrimeScript™ RT reagent Kit with gDNA Eraser (Takara) with random hexamers, following the manufacturer’s instructions. Real-time quantitative PCR was performed using Power SYBR Green PCR Master Mix (Applied Biosystems, 4368708) on the ABI QuantStudio 6 Flex Real-Time PCR System (Applied Biosystems). All primers used for qRT-PCR are listed in Supplementary Table 3.

### Patch-clamp recordings

Patch clamp recordings were performed on *FOXG1*^s/s^ hESCs derived cortical GABA interneurons on day 45 and day 60 of the differentiation. Samples were chosen randomly. Whole-cell current-clamp recordings were performed at 22 °C in artificial cerebral spinal fluid, bubbled with 95% O_2_ and 5% CO_2_. The extracellular fluid consisted of 124 mM NaCl, 3.3 mM KCl, 1.2 mM KH_2_PO_4_, 26 mM NaHCO_3_, 2.4 mM MgSO_4_, 2.5 mM CaCl_2_ and 10 mM glucose (at pH 7.4). TTX (100 nM), or GABA (10 μM) were used in the bath solution. Intracellular solution was containing 135 mM potassium gluconate, 10 mM HEPES, 2 mM MgATP, 7 mM NaCl, 0.3 mM Na_2_GTP and 2 mM MgCl_2_, pH = 7.4. Cell visualization and patch pipette micromanipulation were performed by videomicroscopy, employing a 40× water-immersion objective mounted on an upright microscope equipped with infrared differential interference contrast (Nikon, Eclipse fn1, Japan). Intracellular membrane electrical potentials were recorded in current-clamp mode, using a Multiclamp 700B amplifier (Molecular Devices, Palo Alto, CA, USA). Data was digitized at 10 kHz with a 2 kHz low-pass filters. Data was analyzed using Clampfit 10.6 (Axon Instruments). For voltage clamp recordings, cells were held at −70 mV.

### RNA-seq analysis

RNA samples were shipped to the Beijing Genomics Institute for analysis as a service. The DNBs were loaded into the patterned nanoarrays and pair-end reads of 100 bp were read through on the BGISEQ-500 platform for the following data analysis study. For this step, the BGISEQ-500 platform combines the DNA nanoball-based nanoarrays and stepwise sequencing using Combinational Probe-Anchor Synthesis Sequencing Method.

### Statistical analysis

The number of biological and technical replicates, and parameters are indicated in corresponding figure legends. Data acquisition were performed by investigators blinded to experimental groups. Statistical analysis were performed using GraphPad Prism 6. For all experiments with error bars, data are presented as means ± SEM. The unpaired two-tailed Student’s *t* test was used to calculate statistical significance between two groups. Comparisons among three or more groups, statistical significance were made using one-way ANOVA analysis. The *p* values < 0.05 were considered as significantly different. ****p < 0.0001, ***p < 0.001, **p < 0.01, *p < 0.05.

### Reporting summary

Further information on experimental design is available in the Nature Research Reporting Summary linked to this article.

## Supplementary information

Supplementary Information

Description of Additional Supplementary Files

Supplementary Data 1

Supplementary Data 2

Reporting Summary

Source Data

## Data Availability

All data are available within this study are available from the corresponding authors upon reasonable request. Deposited RNA-seq data, GEO: GSE120079. The raw data underlying Figs. 1d, 1f, 1h, 1j, 1k, 2e, 2f, 3i, 4f, 4k–m, 5n, 6b, 6g–i, 8b are provided as a Source Data file. Uncropped versions of western blots are presented in Supplementary Fig. 10 and Supplementary Fig. 11. The FACS sequential gating strategies are indicated in Supplementary Fig. 12.
