# Supplementary material for: Precisely controlling endogenous protein dosage in hPSCs and derivatives to model FOXG1 syndrome
Source: Nat Commun. 2019 Feb 25;10:928. doi: 10.1038/s41467-019-08841-7 (PMC6389984; doi:10.1038/s41467-019-08841-7)
Supplement: Supplementary file 1 — Supplementary Information [file 41467_2019_8841_MOESM1_ESM.pdf]

Supplementary Information

Precisely controlling endogenous protein dosage in hPSCs and derivatives to model FOXG1 syndrome

Zhu et al.

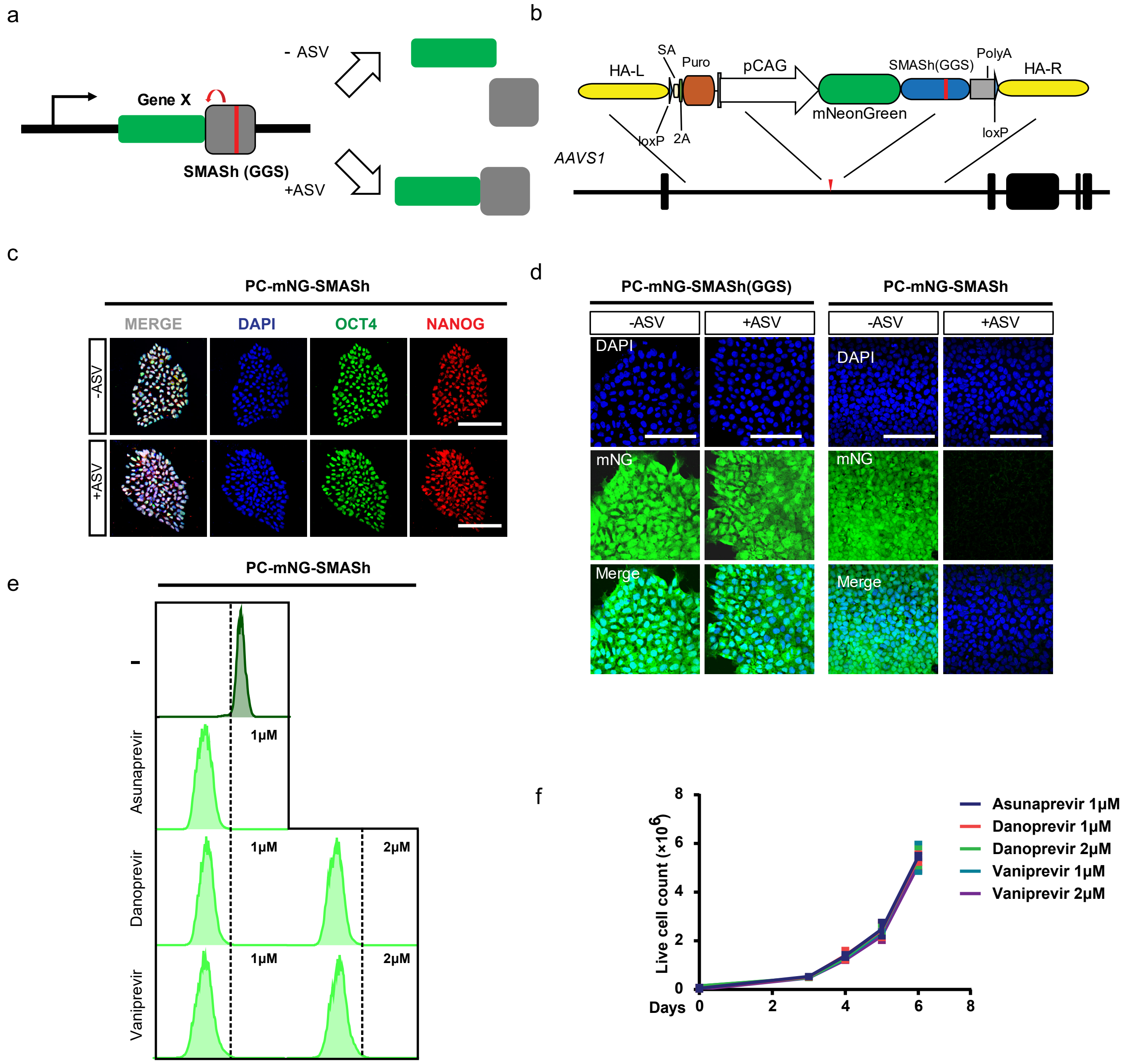
a
- ASV
Gene X
SMASh (GGS)
+ASV
b
HA-L
SA
Puro
pCAG
SMASh(GGS)
PolyA
HA-R
mNeonGreen
loxP
2A
loxP
AAVS1
c
PC-mNG-SMASh
MERGE
DAPI
OCT4
NANOG
-ASV
+ASV
d
PC-mNG-SMASh(GGS)
PC-mNG-SMASh
-ASV
+ASV
-ASV
+ASV
DAPI
mNG
Merge
e
PC-mNG-SMASh
-
Asunaprevir
Danoprevir
Vaniprevir
1μM
1μM
2μM
1μM
2μM
f
Live cell count (×10⁶)
Days
Asunaprevir 1μM
Danoprevir 1μM
Danoprevir 2μM
Vaniprevir 1μM
Vaniprevir 2μM

Supplementary Figure 1. Generation and Characterization of AAVS1-PC-mNG-SMASh hESC line and AAVS1-PC-mNG (GGS)-SMASh hESC line. (**a**) Schematic view of mNG fused mutated SMASh domain fails to induced protein degradation. (**b**) Schematic overview of the strategy for CRISPR/Cas9-mediated targeting to the AAVS1 locus to generate AAVA1-PC-mNeonGreen-SMASh (GGS) hESC line. (**c**) Immunofluorescence images of PC-mNG-SMASh hESCs before and after ASV treatment for pluripotent makers OCT4 (green), NANOG (red). ASV treatment does not compromise pluripotent state of hESCs. Scale bar, 100 μm. (**d**) Immunofluorescence images and comparison of AAVS1-PC-mNG-SMASh and AAVS1-PC-mNG-SMASh (GGS) before and after 4 days ASV (1μm) treatment. ASV successfully induced mNG degradation in AAVS1-PC-mNG-SMASh hESCs rather than AAVS1-PC-mNG-SMASh (GGS) hESCs. Scale bar, 100 μm. (**e**) Flow cytometry analysis of AAVS1-PC-mNG-SMASh hESCs before and after 4 days ASV (1μM) treatment, as well as other NS3 inhibitors like DAV (1 Mm, 2 μM) and VAV (1 μM, 2 μM). (**f**) AAVS1-PC-mNG-SMASh hESCs were cultured with different NS3 inhibitors: ASV (1μM), DAV (1μM, 2 μM) and VAV (1 μM, 2 μM) for 6 days. These NS3 inhibitors do not affect the proliferation hESCs. Error bar represents SEM.

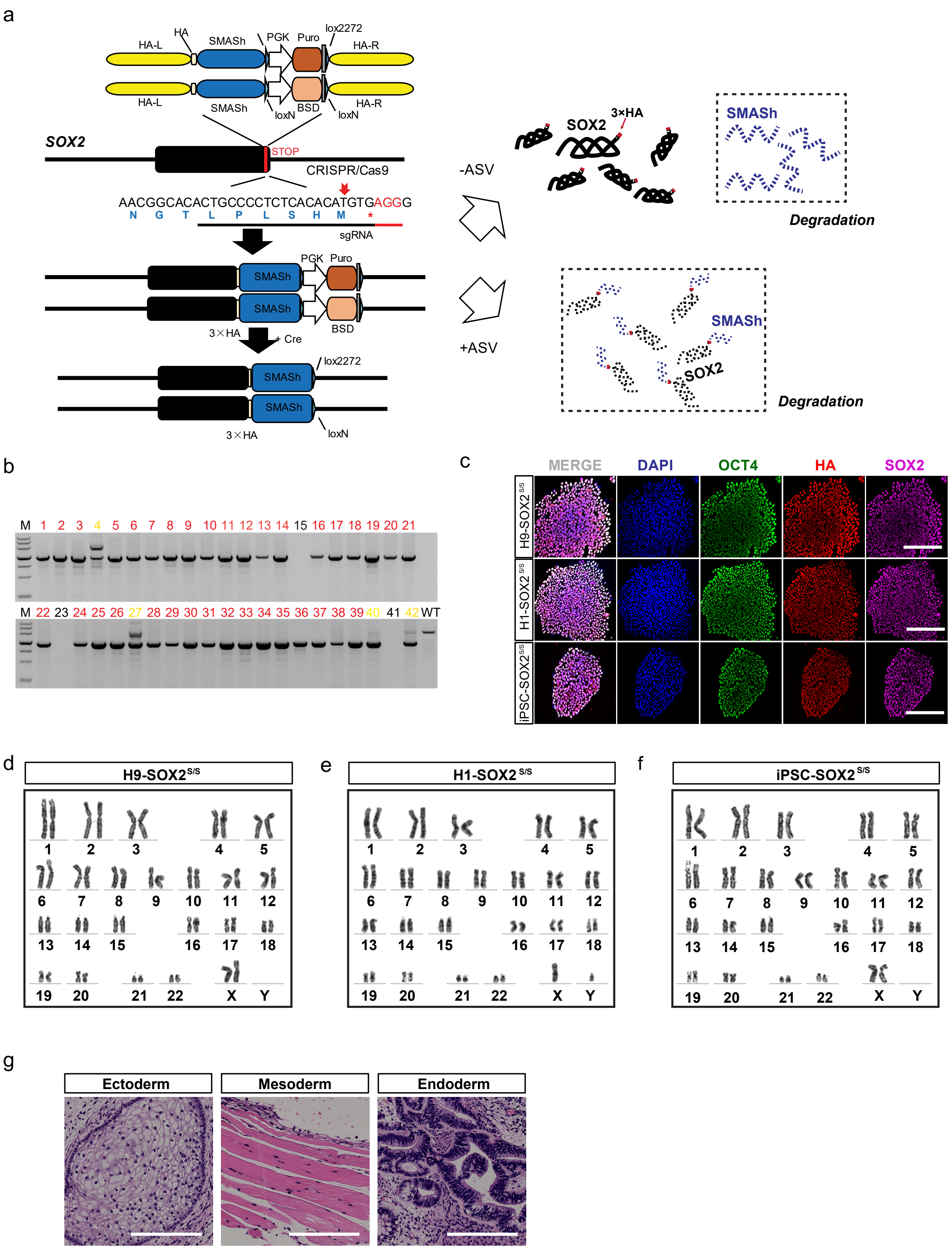

a
HA-L
HA
SMASh
PGK
Puro
lox2272
HA-R
BSD
loxN
SOX2
STOP
CRISPR/Cas9
AACGGCACACTGCCCCTCTCACACATGTGAGGG
N G T L P L S H M *
sgRNA
3×HA
+ Cre
-ASV
+ASV
3×HA
Degradation
b
M 1 2 3 4 5 6 7 8 9 10 11 12 13 14 15 16 17 18 19 20 21
M 22 23 24 25 26 27 28 29 30 31 32 33 34 35 36 37 38 39 40 41 42 WT
c
MERGE
DAPI
OCT4
HA
SOX2
H9-SOX2 S/S
H1-SOX2 S/S
iPSC-SOX2 S/S
d
H9-SOX2 S/S
e
H1-SOX2 S/S
f
iPSC-SOX2 S/S
1 2 3 4 5
6 7 8 9 10 11 12
13 14 15 16 17 18
19 20 21 22 X Y
g
Ectoderm
Mesoderm
Endoderm

Supplementary Figure 2 . Generation and Characterization of SOX2s/s hPSCs. (**a**) Schematic overview of generation of *SOX2*$^{s/s}$ hPSC lines and ASV induced endogenous SOX2 protein degradation. (**b**) PCR genotyping to identify H9SOX2$^{s/s}$ hESC colonies. Rec character indicates homozygous targeted colonies, yellow character indicate heterozygou colonies. (**c**) Immunostaining images of H1 -*SOX2*$^{s/s}$ hESC line, H9 -*SOX2*$^{s/s}$ hESC line, *SOX2*$^{s/s}$ hiPSC line for OCT4 (green), HA (red), SOX2 (magenta). R esults showed coexpression of HA and *SOX2.* Scale bar, 100 μm. (**d-f**) Karyotyping analysis on H9 -SOX2$^{s/s}$ hESCs (**d**), H1-*SOX2*$^{s/s}$ hESCs (**e**), *SOX2* $^{s/s}$ hiPSCs (**f**). (**g**) H&E staining shows ectoderm, mesoderm, endoderm of a teratoma derived from H9 -SOX2$^{s/s}$ hESCs *in vivo.* Scale bar, 200 μm.

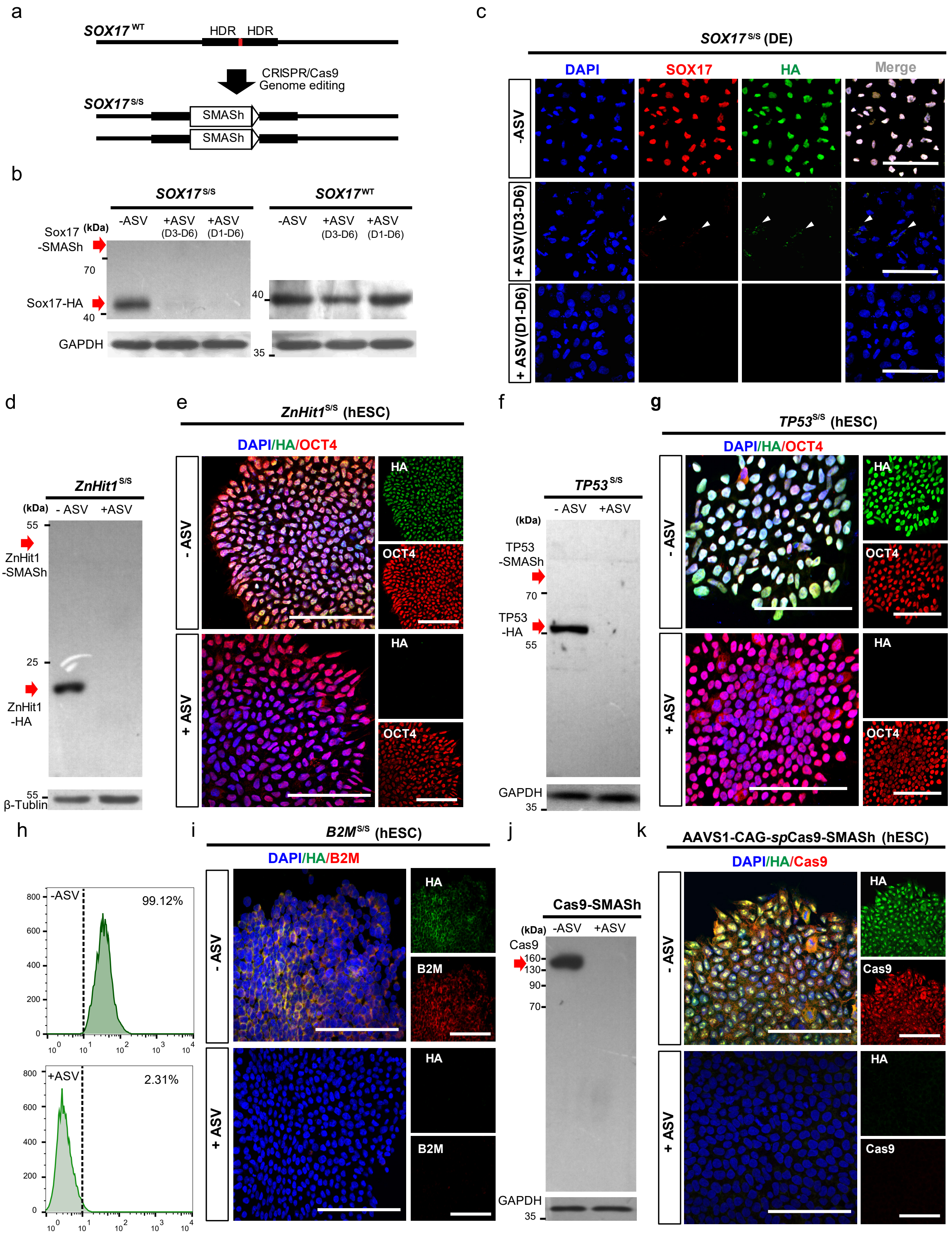
a
SOX17 WT
HDR HDR
CRISPR/Cas9 Genome editing
SOX17 S/S
SMASh
SMASh
b
SOX17 S/S
SOX17 WT
-ASV +ASV (D3-D6) +ASV (D1-D6)
-ASV +ASV (D3-D6) +ASV (D1-D6)
(kDa)
Sox17 -SMASh
70
Sox17-HA
40
GAPDH
35
c
SOX17 S/S (DE)
DAPI
SOX17
HA
Merge
-ASV
+ ASV(D3-D6)
+ ASV(D1-D6)
d
ZnHit1 S/S
(kDa) - ASV +ASV
55
ZnHit1 -SMASh
25
ZnHit1 -HA
55
β-Tublin
e
ZnHit1 S/S (hESC)
DAPI/HA/OCT4
HA
OCT4
- ASV
+ ASV
f
TP53 S/S
(kDa) - ASV +ASV
TP53 -SMASh
70
TP53 -HA
55
GAPDH
35
g
TP53 S/S (hESC)
DAPI/HA/OCT4
HA
OCT4
- ASV
+ ASV
h
-ASV
99.12%
+ASV
2.31%
800
600
400
200
0
i
B2M S/S (hESC)
DAPI/HA/B2M
HA
B2M
- ASV
+ ASV
j
Cas9-SMASh
(kDa) -ASV +ASV
Cas9
160
130
90
70
GAPDH
35
k
AAVS1-CAG-spCas9-SMASh (hESC)
DAPI/HA/Cas9
HA
Cas9
- ASV
+ ASV

Supplementary Figure 3 . Target and Degrade a Broad Range of Endogenous Proteins or Transgenes by SMASh in hPSCs. (**a**) Schematic overview of generation of *SOX17*$^{s/s}$ hPSC lines using CRISPR/Cas9. (**b, c**) Western blot (**b**) and Immunostaining images (**c**) of DE derived from *SOX17* $^{s/s}$ hESCs or WT H9 hESCs after ASV (1 μM) treatment. SOX17 protein production was knocked out in *SOX17*$^{s/s}$ hESCs derived DE. White arrow shows the SOX17 protein aggregates. Scale bar, 100 μm. (**d, f, j**) Western blot of *Znhit1*$^{s/s}$ hESCs (**d**), *TP53* $^{s/s}$ hESCs (**f**), AAVS1-PC-Cas9 (**j**) before and after ASV treatment. (**e, j, i, k**) Immunostaining images of *Znhit1*$^{s/s}$ hESCs (**e**), *TP53* $^{s/s}$ hESCs (**j**), *B2M* $^{s/s}$ hESCs (**i**), AAVS1-PC-Cas9 (**k**) before and after ASV treatment.Scale bar, 100 μm. (**h**) Flow cytometry analysis of *Znhit1*$^{s/s}$ hESCs to detect ZnHit1 expression before and after ASV treatment.

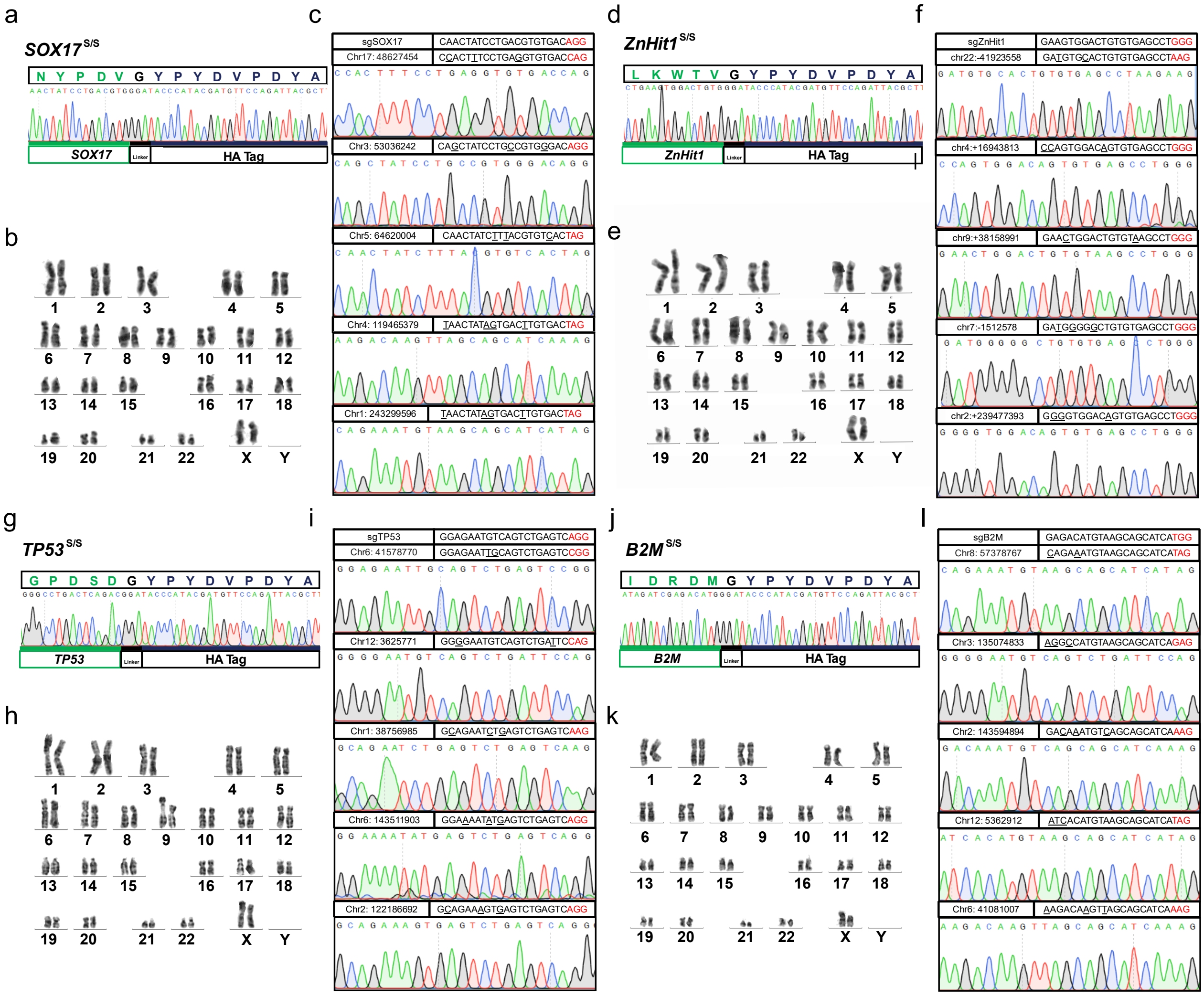

a
SOX17 S/S
SOX17
Linker
HA Tag
b
c
sgSOX17
Chr17: 48627454
Chr3: 53036242
Chr5: 64620004
Chr4: 119465379
Chr1: 243299596
d
ZnHit1 S/S
ZnHit1
Linker
HA Tag
e
f
sgZnHit1
chr22:-41923558
chr4:+16943813
chr9:+38158991
chr7:-1512578
chr2:+239477393
g
TP53 S/S
TP53
Linker
HA Tag
h
i
sgTP53
Chr6: 41578770
Chr12: 3625771
Chr1: 38756985
Chr6: 143511903
Chr2: 122186692
j
B2M S/S
B2M
Linker
HA Tag
k
l
sgB2M
Chr8: 57378767
Chr3: 135074833
Chr2: 143594894
Chr12: 5362912
Chr6: 41081007

Supplementary Figure 4. Characterization and Off Target Analysis of Various SMAS Tagged hESC Lines. (**a, d, g, j**) Representative Sanger sequencing graph of *SOX17* $^{s/s}$ (**a**), *ZnHit1*$^{s/s}$ (**d**), *TP53* $^{s/s}$ (**g**), *B2M* $^{s/s}$ (**j**) hPSC colony showed integration of HA -SMASh fragment. (**b, e, h, k**) Karyotyping analysis on *SOX17*$^{s/s}$ hESCs (**b**), *ZNHIT1*$^{s/s}$ hESCs (**e**), *TP53*$^{s/s}$ hESCs (**h**) and *TP53*$^{s/s}$ hESCs (**k**). (**c, f, i, l**) Representative Sanger sequencing graph of top 5 potential off-target sites of *SOX17*$^{s/s}$ (**c**), *ZnHit1*$^{s/s}$ (**f**), *TP53* $^{s/s}$ (**i**), *B2M* $^{s/s}$ (**l**) hPSC colony showed no off-target mutations. (**m**) Table shows targeted genetic locus anc targeted efficiency for each locus using SMASh.

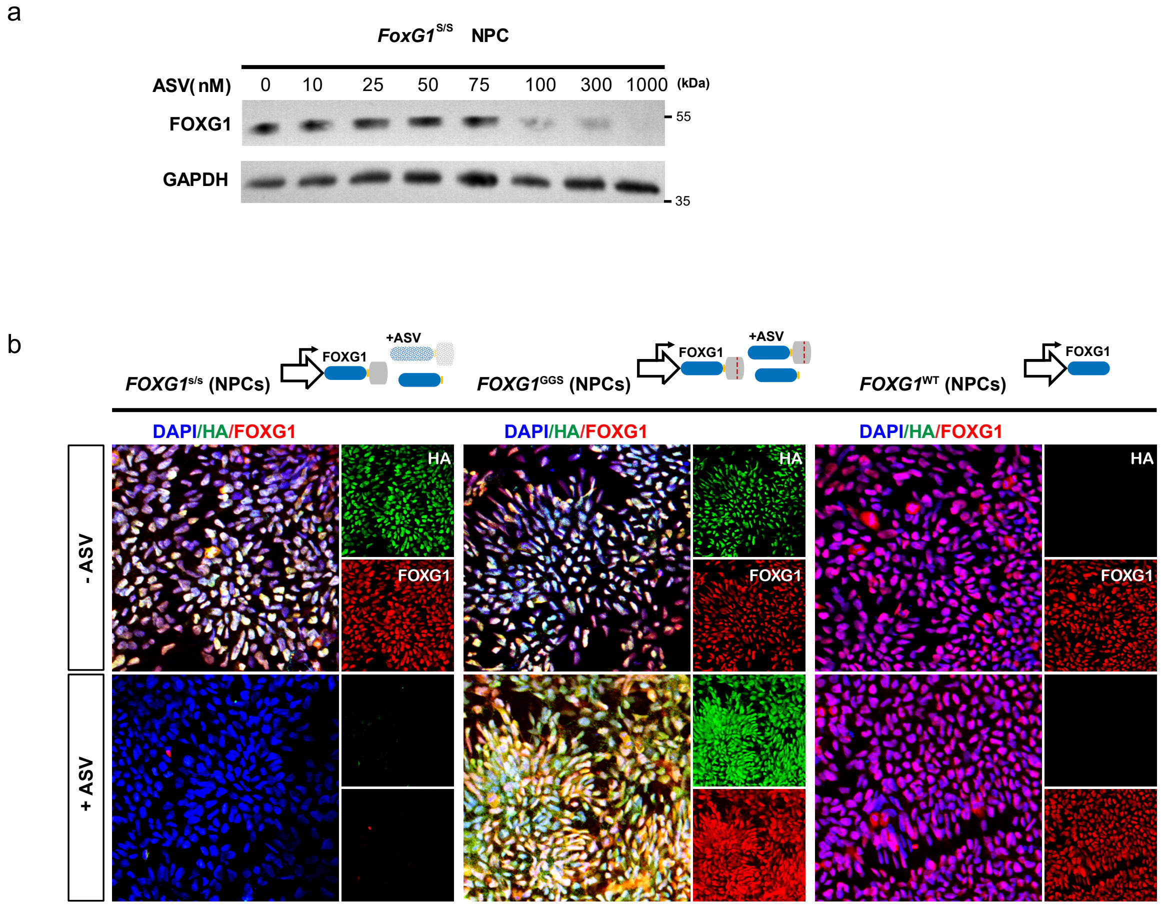
a
FoxG1 S/S NPC
ASV(nM) 0 10 25 50 75 100 300 1000 (kDa)
FOXG1
GAPDH
55
35
b
FOXG1s/s (NPCs)
FOXG1GGS (NPCs)
FOXG1WT (NPCs)
FOXG1
+ASV
DAPI/HA/FOXG1
HA
- ASV
+ ASV

Supplementary Figure 5. S MASh fine-tunes the dosage of FOXG1 protein. (**a**) Western blot of *FOXG1*$^{s/s}$ hESCs under treatment of different concentration of ASV (0 nM 10 nM, 25 nM, 50 nM, 75 nM, 100 nM, 300 nM, 1000 nM) to detect FOXG1 protein . (**b**) Immunofluorescence images of WT hESCs derived NPCs, FOXG1 $^{S/S}$ hESCs derived NPCs and FOXG1-GGS derived NPCs for HA (green), FOXG1 (red). Scale bar, 100 μm.

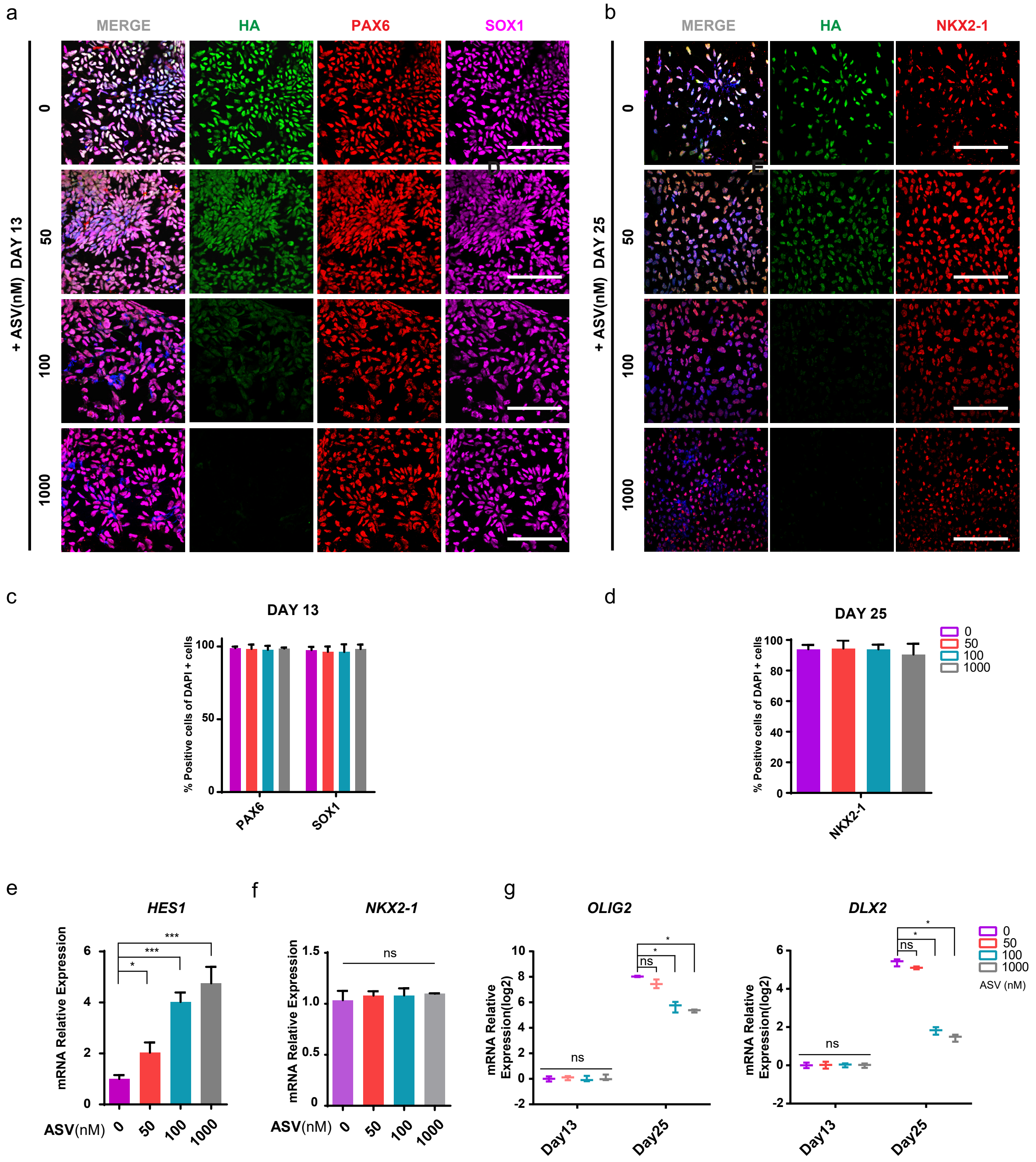

a
MERGE
HA
PAX6
SOX1
+ ASV(nM) DAY 13
0
50
100
1000
b
MERGE
HA
NKX2-1
+ ASV(nM) DAY 25
0
50
100
1000
c
DAY 13
% Positive cells of DAPI + cells
100
50
0
PAX6
SOX1
d
DAY 25
% Positive cells of DAPI + cells
100
80
60
40
20
0
NKX2-1
0
50
100
1000
e
HES1
mRNA Relative Expression
6
4
2
0
*
***
***
ASV(nM)
0
50
100
1000
f
NKX2-1
mRNA Relative Expression
1.5
1.0
0.5
0
ns
ASV(nM)
0
50
100
1000
g
OLIG2
mRNA Relative Expression(log2)
10
8
6
4
2
0
-2
ns
ns
*
*
Day13
Day25
DLX2
mRNA Relative Expression(log2)
6
4
2
0
-2
ns
ns
*
*
Day13
Day25
0
50
100
1000
ASV (nM)

Supplementary Figure 6. FOXG1 Regulates the Proliferation of Telencephalic Progenitors and Cortical GABA Interneuron Development in a Dose Dependent Manner. (**a, c**) Immunofluorescence images (**a**) and quantification (**c**) of FOXG1-expressing telencephalic progenitors (day 13) for HA (green), PAX6 (red), SOX1 (magent under the treatment of different concentration of ASV (0 nM, 50 nM, 100 nM, 1000 nM), showed dosage regulation of FOXG1 protein. Scale bar, 100 μ m. (**b, d**) Immunofluorescence images (**b**) and and quantification (**d**) of FOXG1-expressing medial ganglionic eminence (MGE, day 25) progenitors for HA (green), *NKX2-1* (red) under the treatment of different concentration of ASV (0 nM, 50 nM, 100 nM, 1000 nM). Scale bar, 100 μm. (**e, f**) qPCR analysis on day 25 (n=3) under treatment of ASV (0 nM, 50 nM, 100 nM, 1000 nM) for *HES1* (**e**) and *NKX2-1* (**f**). (**g**) qPCR analysis on day 13 and day 25 (n=3) under treatment of ASV (0 nM, 50 nM, 100 nM, 1000 nM) for *OLIG2* and *DLX2*. Error bar represents SEM (*p < 0.05, **p < 0.01, ***p < 0.001, **** p < 0.0001).

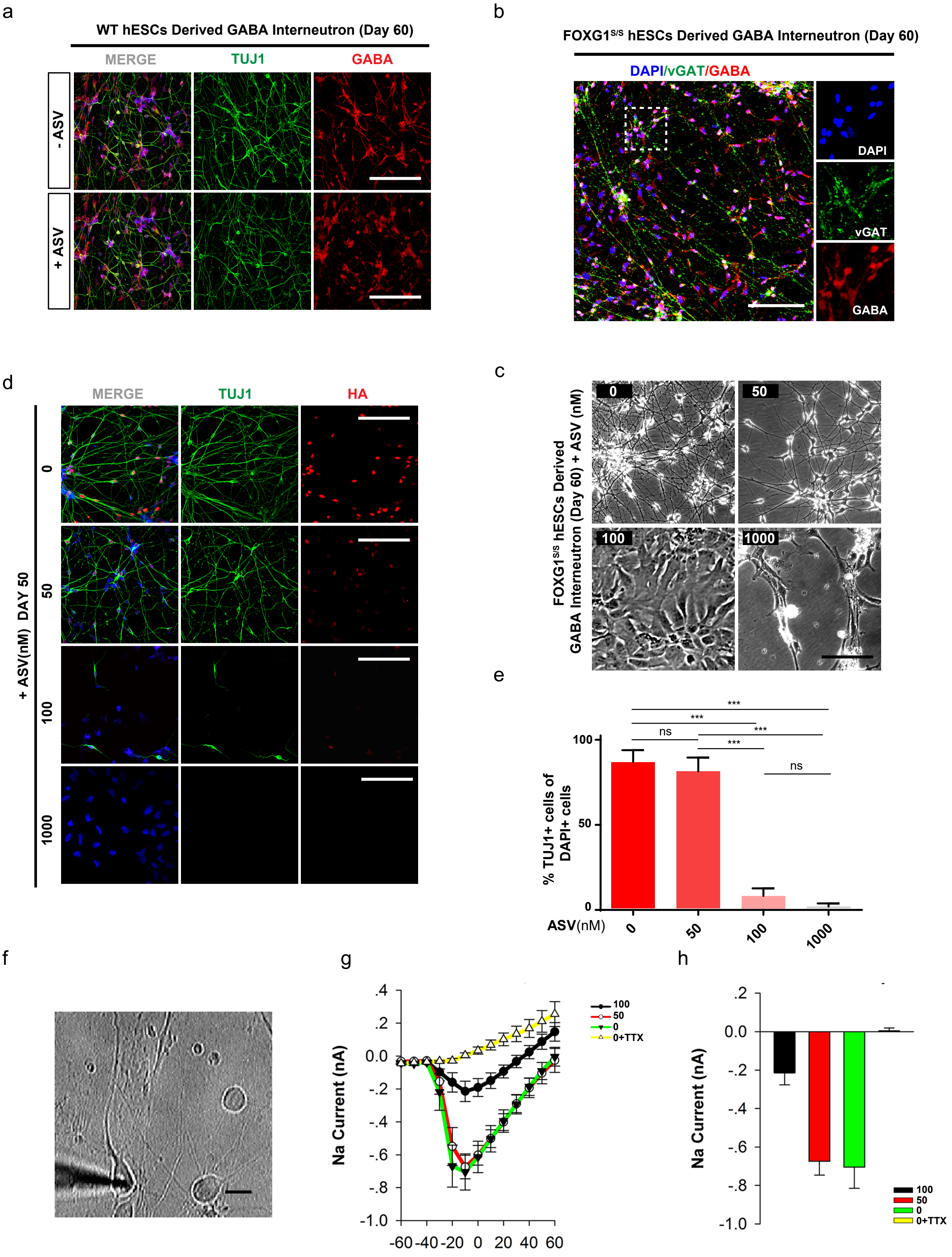

a
WT hESCs Derived GABA Interneutron (Day 60)
MERGE
TUJ1
GABA
- ASV
+ ASV
b
FOXG1S/S hESCs Derived GABA Interneutron (Day 60)
DAPI/vGAT/GABA
DAPI
vGAT
GABA
d
MERGE
TUJ1
HA
+ ASV(nM) DAY 50
0
50
100
1000
c
FOXG1S/S hESCs Derived GABA Interneutron (Day 60) + ASV (nM)
0
50
100
1000
e
% TUJ1+ cells of DAPI+ cells
100
50
0
***
***
***
ns
***
ns
ASV(nM)
0
50
100
1000
f
g
Na Current (nA)
.4
.2
0.0
-.2
-.4
-.6
-.8
-1.0
-60 -40 -20 0 20 40 60
100
50
0
0+TTX
h
Na Current (nA)
.2
0.0
-.2
-.4
-.6
-.8
-1.0
100
50
0
0+TTX

Supplementary Figure 7 . FOXG1 Insufficiency Block Cortical GABA Interneuron Induction and Maturation. (**a**) Immunofluorescence images of WT hESCs derived cortica[illegible] GABA interneuron (day 60) for TUJ1 (green), GABA (red) with or without ASV treatment ([illegible] μM). (**b**) Immunofluorescence images of *FOXG1*$^{S/S}$ hESCs derived cortical GABA interneuron (day 60) for vGAT (green), GABA (red), showed colocalized GABA and vGAT[illegible] expression Scale bar, 100 μm. (**c**) Representative images of GABA interneurons from eac[illegible] ASV treatment group (0 nM, 50 nM, 100 nM, 1000 nM) on day 60. Scale bar, 100 μm. (**d, e**) Immunofluorescence images (**d**) and quantification (**e**) on day 60 for TUJ1 (green), FOXG1-*HA* (red) under the treatment of different concentration of ASV (0nM, 50nM, 100n[illegible] 1000nM). Scale bar, 100 μm. (**f**) A DIC image of *FOXG1*$^{S/S}$ hESCs derived neurons on da[illegible] 60. (**g, h**) Voltage-current relationships for Na+ currents on day 60. Error bar represents SEM (*p < 0.05, **p < 0.01, ***p < 0.001, **** p < 0.0001).

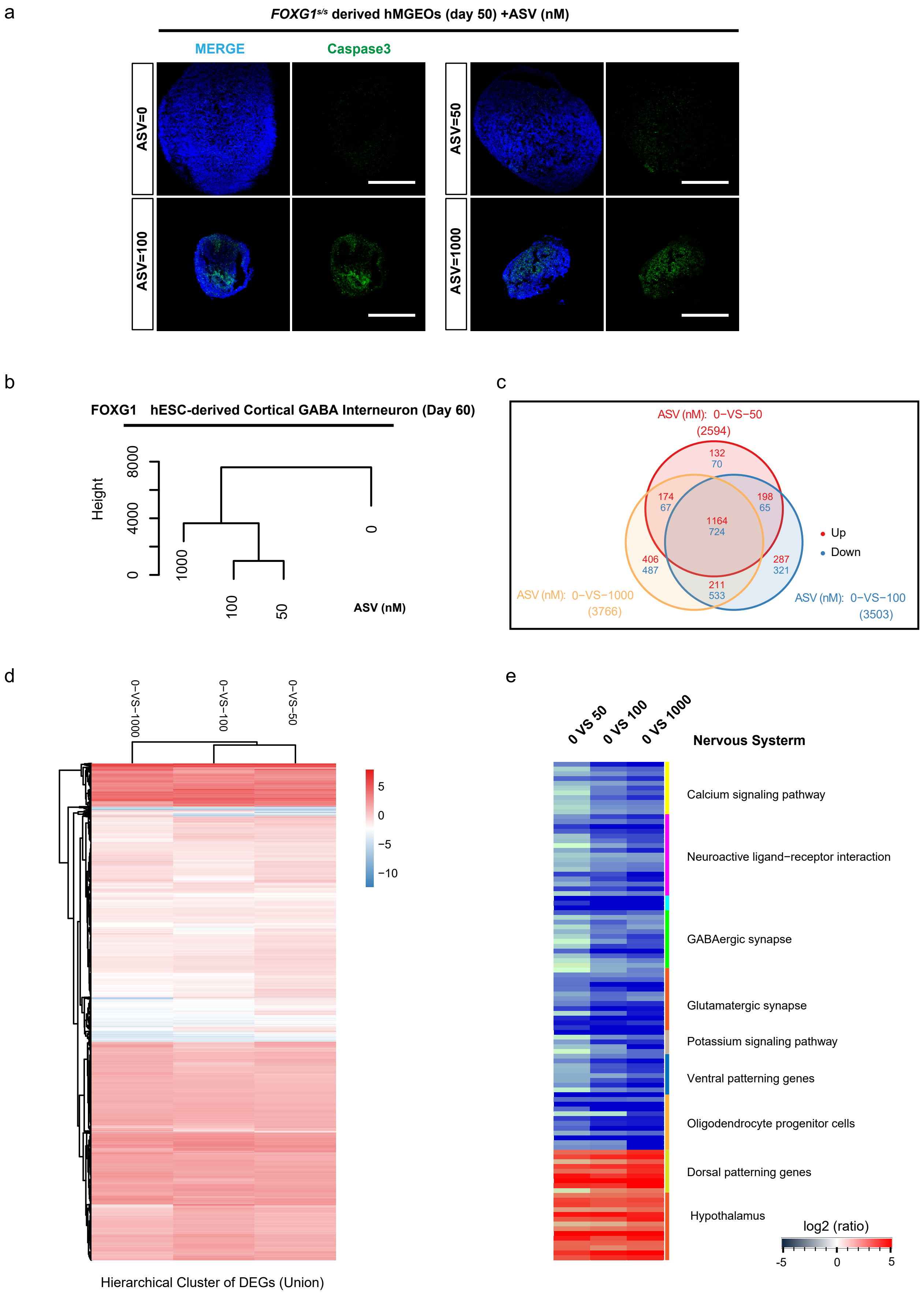

a
FOXG1s/s derived hMGEOs (day 50) +ASV (nM)
MERGE
Caspase3
ASV=0
ASV=50
ASV=100
ASV=1000
b
FOXG1 hESC-derived Cortical GABA Interneuron (Day 60)
Height
0
4000
8000
1000
100
50
0
ASV (nM)
c
ASV (nM): 0−VS−50
(2594)
132
70
174
67
198
65
1164
724
406
487
287
321
211
533
Up
Down
ASV (nM): 0−VS−1000
(3766)
ASV (nM): 0−VS−100
(3503)
d
0−VS−1000
0−VS−100
0−VS−50
5
0
−5
−10
Hierarchical Cluster of DEGs (Union)
e
0 VS 50
0 VS 100
0 VS 1000
Nervous Systerm
Calcium signaling pathway
Neuroactive ligand−receptor interaction
GABAergic synapse
Glutamatergic synapse
Potassium signaling pathway
Ventral patterning genes
Oligodendrocyte progenitor cells
Dorsal patterning genes
Hypothalamus
log2 (ratio)
-5
0
5

Supplementary Figure 8. RNA-seq Analysis of Derived Cortical GABA Interneurons. (**a**) Immunostaining against Caspase3 (green) on FOXG1S/S hESCs derived hMGEOs from each ASV treatment group (0 nM, 50 nM, 100 nM, 1000 nM). Scale bar, 500 μm. (**b**) Hierarchical clustering between sampl es. (**c**) Venn diagram of DEGs. The red number represents the up -regulated gene amount, blue number represents the down -regulated gene amount. (**d)** Heatmap of hierarchical clustering of DEGs. Coloring indicates the log2 transformed fold change. Union indicatethe file contents are intersection or union of DEGs among different comparison groups. (**e**) Expression profile of DEGs related to variety of synapses formation. Coloring indicates the log2 transformed fold change.

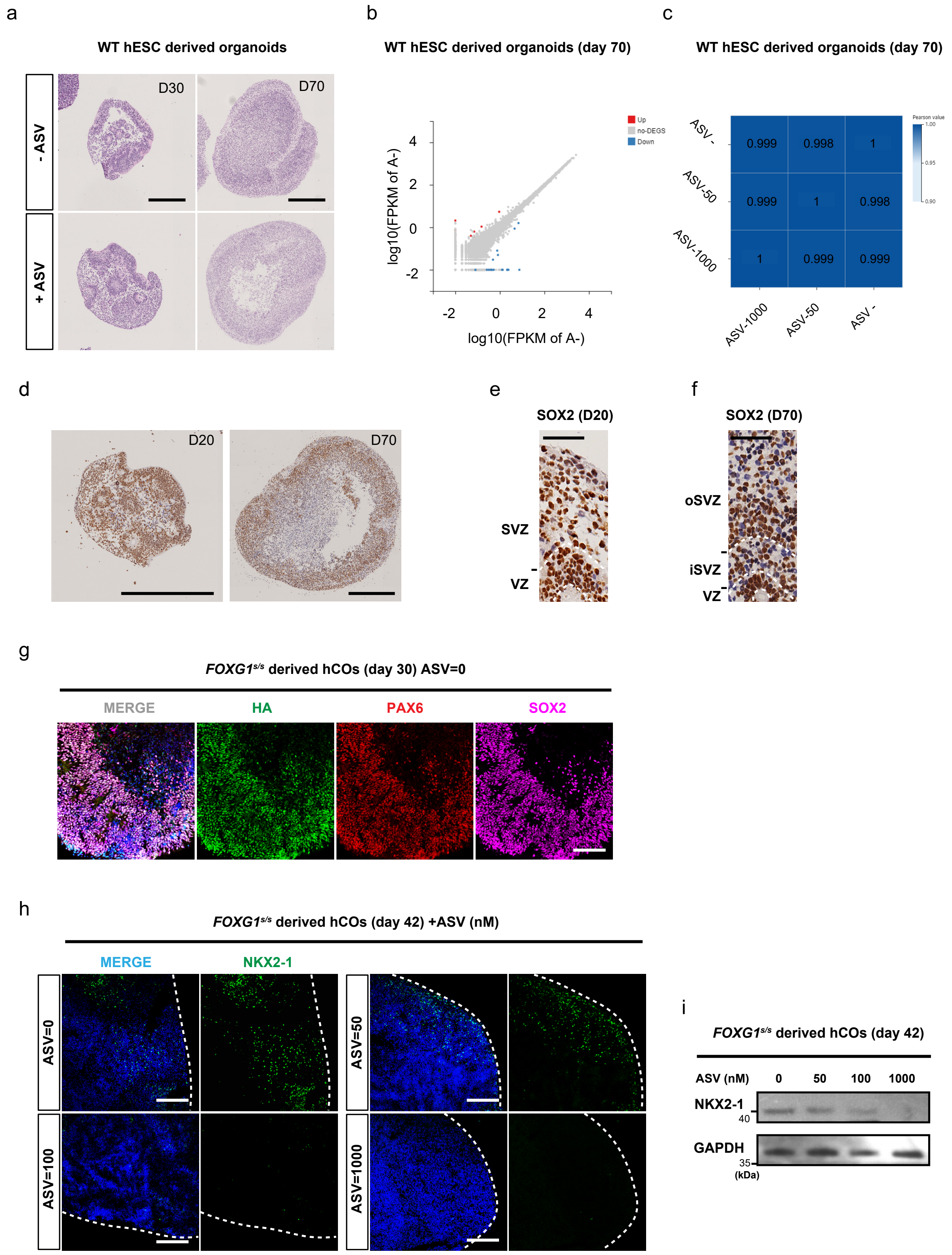
a
WT hESC derived organoids
- ASV
+ ASV
D30
D70
b
WT hESC derived organoids (day 70)
log10(FPKM of A-)
Up
no-DEGS
Down
c
WT hESC derived organoids (day 70)
ASV -
ASV-50
ASV-1000
0.999
0.998
1
0.999
1
0.998
1
0.999
0.999
Pearson value
d
D20
D70
e
SOX2 (D20)
SVZ
VZ
f
SOX2 (D70)
oSVZ
iSVZ
VZ
g
FOXG1s/s derived hCOs (day 30) ASV=0
MERGE
HA
PAX6
SOX2
h
FOXG1s/s derived hCOs (day 42) +ASV (nM)
MERGE
NKX2-1
ASV=0
ASV=50
ASV=100
ASV=1000
i
FOXG1s/s derived hCOs (day 42)
ASV (nM)
0
50
100
1000
NKX2-1
40
GAPDH
35
(kDa)

Supplementary Figure 9. Model FOXG1 syndrome in hPSC -derived cortical organoids using SMASh. (**a**) Haematoxylin and eosin (HE) staining images of WT hESC derived cortical organoids on day 20 and day 70 with or without ASV treatment. Scale bar 500 μm. (**b**) MA plots chart of differentially expressed genes (DEGs) with or without ASV treatment. Red, blue and gray dots represent up -regulated, down-regulated DEGs and non-DEGs. (**c**) Correlation heatmap showing correlation between grou p with or without ASV treatment. (**d**) Immunostaining image for SOX2 of hMGEOs on day 20 and on day 70 Scale bar, 500 μm. (**e**) Immunostaining image for SOX2 of hMGEOs on day 20. Dashed lines highlight a gap between VZ and SVZ zone. Scale bar, 100 μm. (**f**) Immunostaining image for SOX2 of hMGEOs on day 70, showing the diminishment of VZ-like area. Dashed lines highlight gaps among VZ, iSVZ and oSVZ zone. Scale bar, 100 μm. (**g**) Representative Immunostaining images of *FOXG1*$^{S/S}$ hESCs derived hCOs on day 30 for PAX6 (red), SOX2 (magenta), HA (green). Scale bar, 100 μm. (**h**) Representative Immunostaining images of *FOXG1*$^{S/S}$ hESCs derived hCOs on day 42 for NKX2-1 (green). Scale bar, 200 μm. (**i**) Western blot of *FOXG1*$^{s/s}$ hESCs derived hCOs on day 42 to detect ventral forebrain maker NKX2-1, showed decreasing of NKX2-1 protein level, as shutting off FOXG1 protein production.

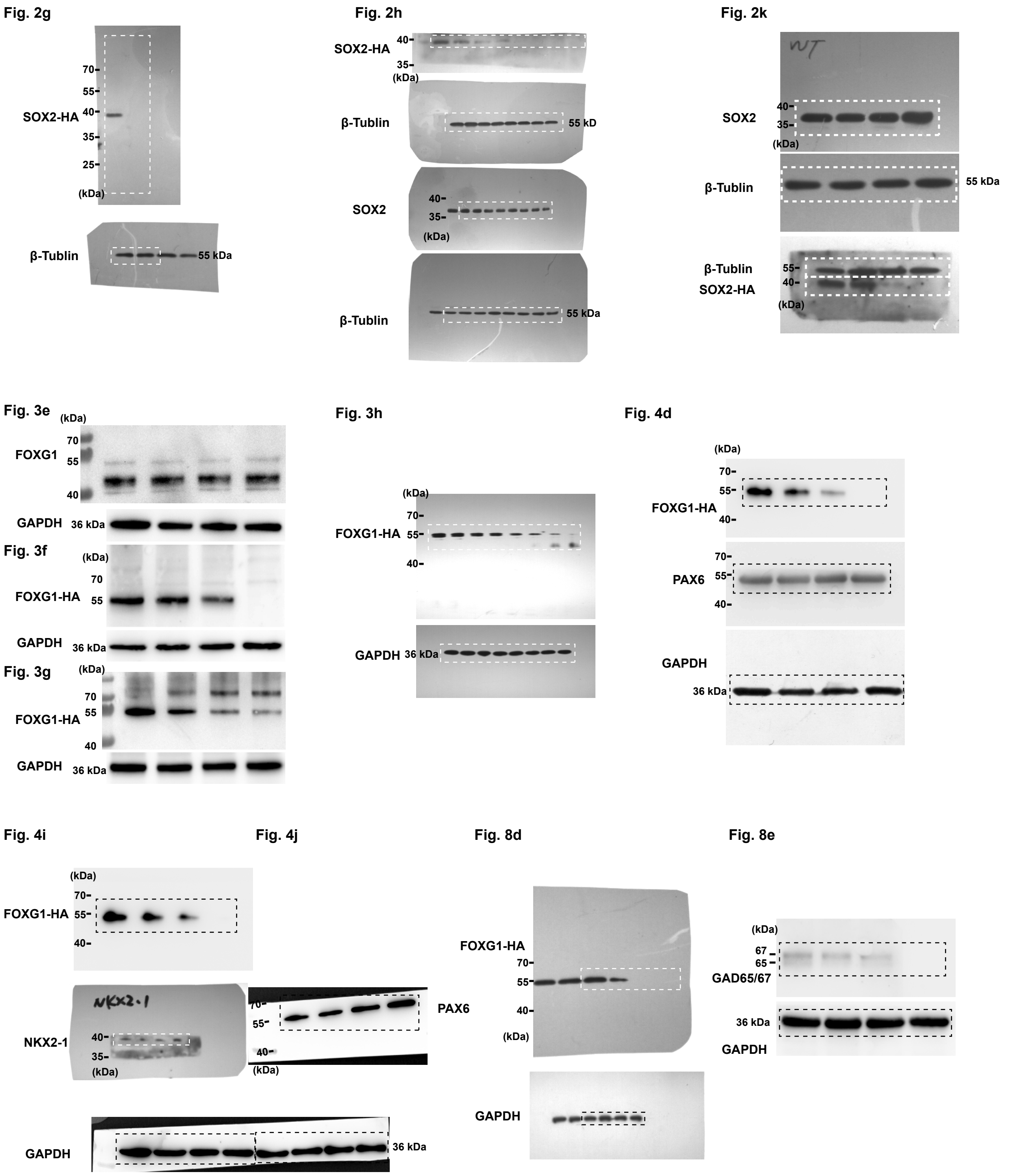

Supplementary Figure 10. Uncropped images of western blots in main figures.

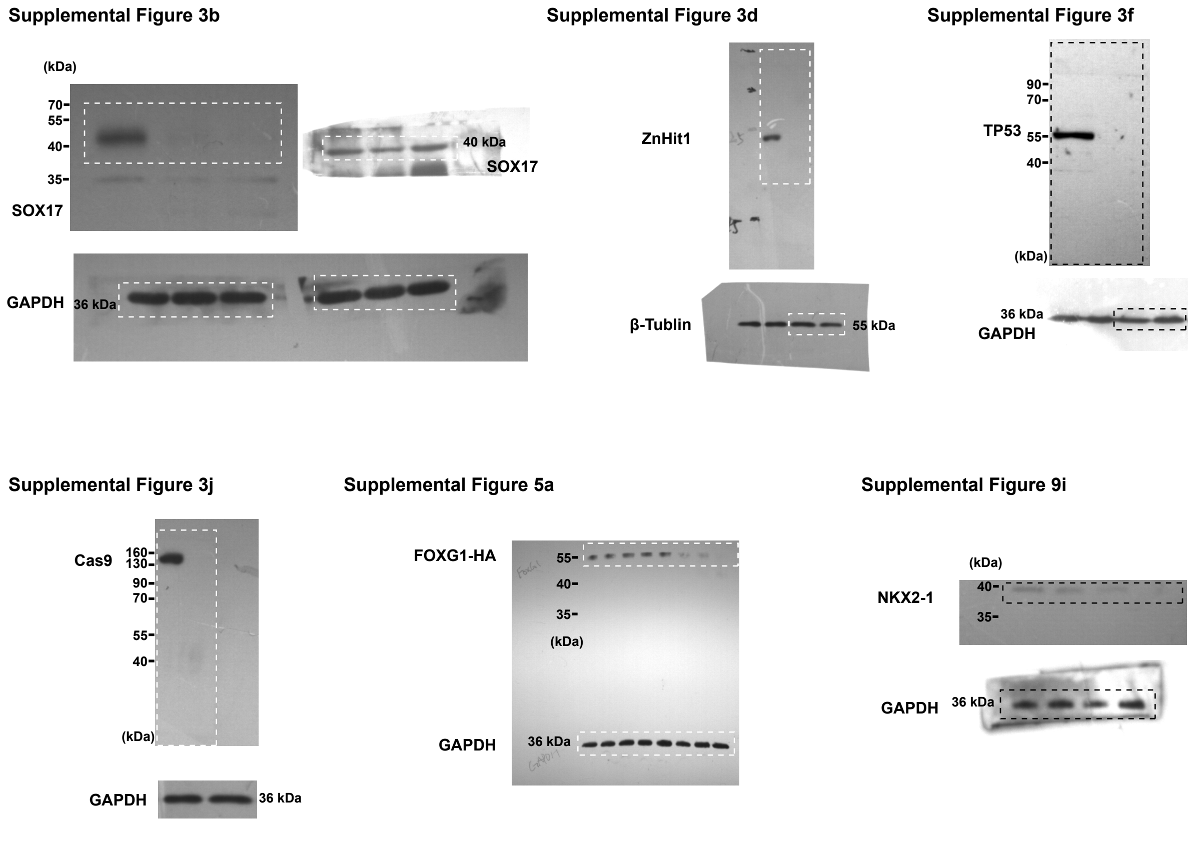

Supplementary Figure 11. Uncropped images of western blots in supplemental figures.

Fig. 1c, e

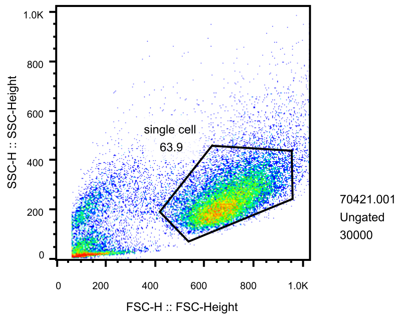

Fig. 1i

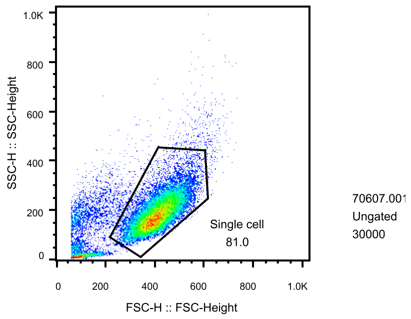

Supplemental Figure 1e

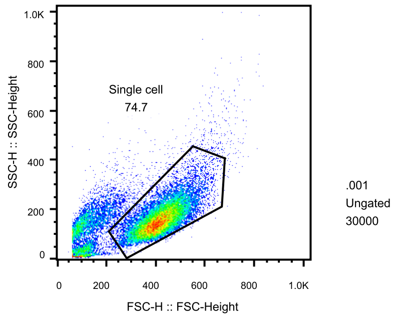

Supplemental Figure 3h

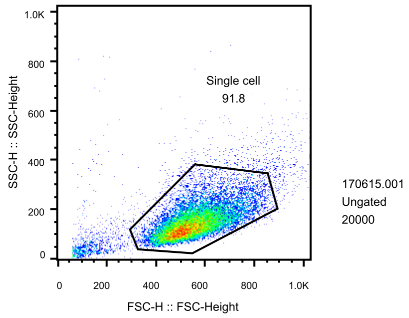

Fig. 4e

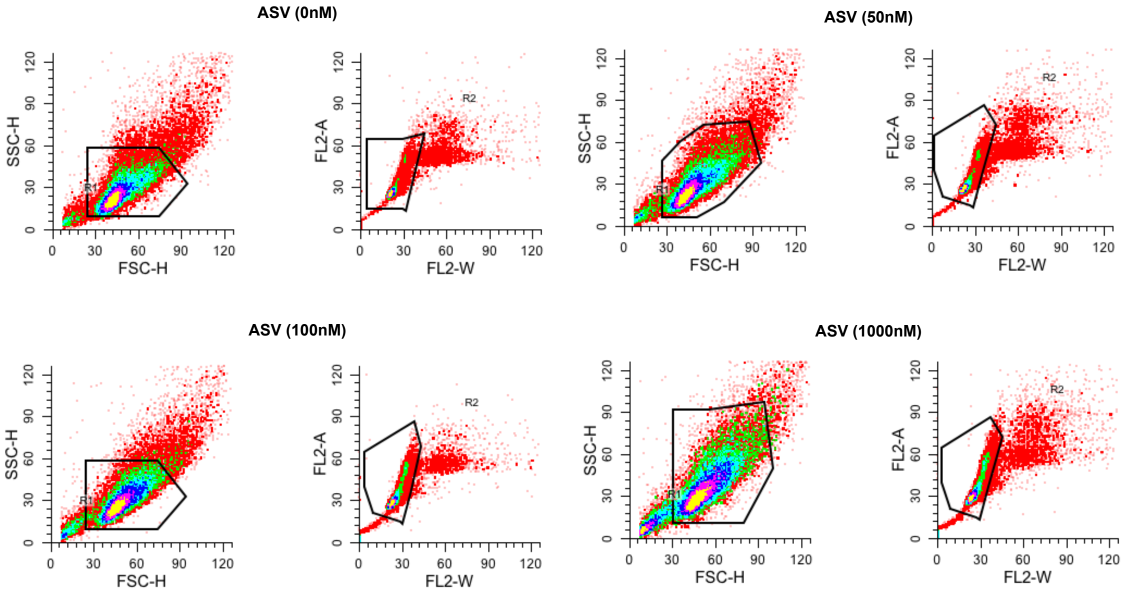

Supplementary Figure 12. All FACS sequential gating strategies in figures.

Supplementary Table 1

Primers for Off-target Analysis

B2M-OT1-F:	TTTCCTGAAGTCTTTCTCCTCG	B2M-OT1-R:	CTAAAATGGCAATGCTGATGCCTG
B2M-OT2-F:	CCTCAGTTTCCCCATATGAACATG	B2M-OT2-R:	AGCTGCTTGTTGGATTGTGCATGG
B2M-OT3-F:	CTGATATGTTGATTGCTGGTGGAG	B2M-OT3-R:	TCCGGGGATGCAAGGACCATTG
ZnHit1-OT4-F:	AGGTGTGCTGATGCTCTGTCAGG	ZnHit1-OT4-R:	GACAACAGTGACCTGGTGATGCTG
ZnHit1-OT5-F:	ATAGCCACACCGGGCTGTGCTCC	ZnHit1-OT5-R:	ATGAAGAGTGAGTTAGTTTCGACAG
SOX17-OT1-F:	TTCTCCACTGAATAACCACCCAGG	SOX17-OT1-R:	AGAGCAGGAGCCTCAGGTGAG
SOX17-OT2-F:	TAAAACATAGAGCATGCCCTCAAAG	SOX17-OT2-R:	AATGAAATCTTTCTAGGAAGCCCG
SOX17-OT3-F:	AATAGTACACATGTACTTTCCTGTTG	SOX17-OT3-R:	ACAATTCCAAAGATCTCTACTAATTG
SOX17-OT4-F:	CCAAAATAATACGGAAACTTTGACAAG	SOX17-OT4-R:	GCAGATTATCTCCCATGACCTG
SOX17-OT5-F:	GATTGTCTTATCTTCTTAGAGAACTG	SOX17-OT5-R:	GACCAAAATAATACGGAAACTTTGACAAG
TP53-OT1-F:	CTTGATGTCTCTCTGTCTCTTTTG	TP53-OT1-R:	AATTCTGAGCCTCCCTCTGCTGG
TP53-OT2-F:	CTAACAAAATTGTAGCTGACCTCCA	TP53-OT2-R:	TTGTTGTTGGCAGCACCAAGCAG
TP53-OT3-F:	ATTCTCCCAGGAGAAACATCAGAG	TP53-OT3-R:	CTGAGGGAGCTCCAGCTCCAG
TP53-OT4-F:	GGCGTTCCGAATGACCTCAG	TP53-OT4-R:	CACGCTTCGGACGTGTGGAG
TP53-OT5-F:	TGGGAGCTGGAGGTTGCAATAAG	TP53-OT5-R:	AGAGAGATGGCTTAAGTGGTTCTG
FOXG1-OT1-F	CATCTACATTGGTATCAAATATCAGG	FOXG1-OT1-R	TGAGTCCTATCTAGGTCTATATAAG
FOXG1-OT2-F	CTACTCTGTTGTGATTTCTGCTGG	FOXG1-OT2-R	ATCACAATTCAAGTTACATCAGGG
FOXG1-OT3-F	CCAGGTGGAGAATATTTGTATGG	FOXG1-OT3-R	GAAACACAGATCACTTCTGTGG
FOXG1-OT4-F	TAAATGGTTAATGAGACATGCC	FOXG1-OT4-R	TCTGCATGTAGTACTCAGAAAGG
FOXG1-OT5-F	CTGGTCTTTAAAGCTCCATCCTG	FOXG1-OT5-R	ACAAATACCTGCGTGATTTCCCAG
SOX2-OT1-F	ACCTCCGGGACCTGATCAGCACG	SOX2-OT1-R	TCCATGCTGTTTCCTACTCTCCTC
SOX2-OT2-F	GGAGCAATCACAGCTTTCTACTTCC	SOX2-OT2-R	TAAATGTATTTCATCATTAAGGC
SOX2-OT3-F	GCTCGATACATTGGCATGGTATAG	SOX2-OT3-R	TTCAGCGTTTAAAGCACATCCTCTG
SOX2-OT4-F	GCTGCAACCACTCTTGGCATTCC	SOX2-OT4-R	AGAGTAACTGTCCAACGGGCATG
SOX2-OT5-F	TCTTGGGTGTTCCCTTAACCTTGG	SOX2-OT5-R	TCACCTGATTGACGACAAAGGTGG

Supplementary Table 2

Primers for Genotyping of SMASh Tagged hESC Colonies.

B2M-seq-F:	TTTCCTGAATTGCTATGTGTCTGGG	B2M-seq-R:	TCTGTTTCCCTTTATCTCCTCTAGTG
FOXG1-seq-F:	CAAGTACGAGAAGCCGCCGTTCAG	FOXG1-seq-R:	CACAACACAAACTGAAGGCAATCG
SOX2-seq-F:	AGCGCCCGCATGTACAACATGATG	SOX2-seq-R:	GCAACTGTCCTAAATTTCAGCTGCAG
SOX17-seq-F:	AGAAGCGGCCCTTCGTGGAGGAG	SOX17-seq-R:	CCTTCTACTTAAAGTGATATACTGG
TP53-seq-F:	GAATCCTATAACCACATTCTTGCCTC	TP53-seq-R:	TCTACCTAACCAGCTGCCCAACTG
ZnHit1-seq-F:	TCTGCAGAACTTGAGTGTGGCCGAG	ZnHit1-seq-R:	GAAACTTCTGGGTTTTCCGTACTG
AAVS1-seq-F:	TCGACCTACTCTCTTCCGCATTGG	AAVS1-seq-R:	CGCACCGTGGGCTTGTACTCGG
SMASh-seq-R:	GGAGCCCTTATCATCGTCGTCCTTG		

Supplementary Table 3

Primers for qRT-PCR.

RT-LHX9-F:	GGGAGTGGACATCGTCAATTA	RT-LHX9-R:	GTCTTCTGCGAGGGTGGATA
RT-HES1-F:	CGGACATTCTGGAAATGACA	RT-HES1-R:	TACTTCCCCAGCACACTTGG
RT-PAX6-F:	TCCGTTGGAACTGATGGAGT	RT-PAX6-R:	GTTGGTATCCGGGGACTTC
RT-NKX2-1-F:	TGAACGTAAGATGGAGGAACA	RT-NKX2-1-R:	GCCAAGGTCAGCCTTATTGA
RT-DLX2-F:	AGCAGCTATGACCTGGGCTA	RT-DLX2-R:	AATTTCAGGCTCAAGGTCCT
RT-ASCL1-F:	CTAAAGATGCAGGTTGTGC	RT-ASCL1-R:	GGAGCTTCTCGACTTCACCA
RT-SIX6-F:	CTGTGACAGGACCTGCTGC	RT-SIX6-R:	CAACCGGACTGACCCCTAC
RT-OLIG2-F:	GATAGTCGTCGCAGCTTTCG	RT-OLIG2-R:	CCTGAGGCTTTTCGGAGC
RT-SST-F:	TGGGTTCAGACAGCAGCTC	RT-SST-R:	CCCAGACTCCGTCAGTTTCT
RT-GAD1-F:	GCGGACCCCAATACCACTAAC	RT-GAD1-R:	CACAAGGCGACTCTTCTCTTC
RT-PDGFRA-F:	TTGAAGGCAGGCACATTTACA	RT-PDGFRA-R:	GCGACAAGGTATAATGGCAGAAT
RT-MYT1-F:	GAAGGAGCCCCGTCAAGTC	RT-MYT1-R:	AGAAGTTGCGATGATTCCCTG
RT-SOX10-F:	CCTCACAGATCGCCTACACC	RT-SOX10-R:	CATATAGGAGAAGGCCGAGTAGA
RT-NPY2R-F:	CATCTTGCTTGGGGTAATTGGC	RT-NPY2R-R:	AGAGTGAACGGTAGACACAGAG
RT-RAX-F:	AAGCCCCTCGACCCTACTG	RT-RAX-R:	CCGCCGATGCTTTTTCTTGG
RT-OTP-F:	GCACAGCTCAACGAGTTGGA	RT-OTP-R:	GTCAGCCCGATACGCAGTG
RT-PMCH-F:	TTTCAAAGAACACAGGCTCCAAA	RT-PMCH-R:	GCATACATCTGAGCATGTCAAAATC
RT-GAPDH-F:	CGGATTTGGTCGTATTGGG	RT-GAPDH-R:	CGCTCCTGGAAGATGGTGAT
