# Supplementary material for: Precisely controlling endogenous protein dosage in hPSCs and derivatives to model FOXG1 syndrome
Source: Nat Commun. 2019 Feb 25;10:928. doi: 10.1038/s41467-019-08841-7 (PMC6389984; doi:10.1038/s41467-019-08841-7)
Supplement: Supplementary file 2 — Description of Additional Supplementary Files [file 41467_2019_8841_MOESM2_ESM.docx]

**Description of Additional Supplementary Files**

File Name: Supplementary Data 1

Description: Antibodies and key reagents used for this study.

File Name: Supplementary Data 2

Description: DNA sequence of main plasmids used for this study.
