# Supplementary material for: Precisely controlling endogenous protein dosage in hPSCs and derivatives to model FOXG1 syndrome
Source: Nat Commun. 2019 Feb 25;10:928. doi: 10.1038/s41467-019-08841-7 (PMC6389984; doi:10.1038/s41467-019-08841-7)
Supplement: Supplementary file 5 — Reporting Summary [file 41467_2019_8841_MOESM5_ESM.pdf]

nature research

Corresponding author(s): Qi Zhou, Wei Li, Baoyang Hu

Reporting Summary

Nature Research wishes to improve the reproducibility of the work that we publish. This form provides structure for consistency and transparency in reporting. For further information on Nature Research policies, see Authors & Referees and the Editorial Policy Checklist.

Statistical parameters

When statistical analyses are reported, confirm that the following items are present in the relevant location (e.g. figure legend, table legend, main text, or Methods section).

n/a	Confirmed	
☐	☒	The exact sample size (*n*) for each experimental group/condition, given as a discrete number and unit of measurement
☐	☒	An indication of whether measurements were taken from distinct samples or whether the same sample was measured repeatedly
☐	☒	The statistical test(s) used AND whether they are one- or two-sided<br>*Only common tests should be described solely by name; describe more complex techniques in the Methods section.*
☒	☐	A description of all covariates tested
☐	☒	A description of any assumptions or corrections, such as tests of normality and adjustment for multiple comparisons
☐	☒	A full description of the statistics including central tendency (e.g. means) or other basic estimates (e.g. regression coefficient) AND variation (e.g. standard deviation) or associated estimates of uncertainty (e.g. confidence intervals)
☐	☒	For null hypothesis testing, the test statistic (e.g. *F*, *t*, *r*) with confidence intervals, effect sizes, degrees of freedom and *P* value noted<br>*Give P values as exact values whenever suitable.*
☒	☐	For Bayesian analysis, information on the choice of priors and Markov chain Monte Carlo settings
☒	☐	For hierarchical and complex designs, identification of the appropriate level for tests and full reporting of outcomes
☒	☐	Estimates of effect sizes (e.g. Cohen's *d*, Pearson's *r*), indicating how they were calculated
☐	☒	Clearly defined error bars<br>*State explicitly what error bars represent (e.g. SD, SE, CI)*

Our web collection on statistics for biologists may be useful.

Software and code

Policy information about availability of computer code

Data collection	Confocal images were collected using ZEN 2012.
Data analysis	We used the following software: Flowjo-v10, GraphPad Prism v6, VennDiagram, R, pheatmap, ggplot2, ModFitLT V3.2.

For manuscripts utilizing custom algorithms or software that are central to the research but not yet described in published literature, software must be made available to editors/reviewers upon request. We strongly encourage code deposition in a community repository (e.g. GitHub). See the Nature Research guidelines for submitting code & software for further information.

Data

Policy information about availability of data

All manuscripts must include a data availability statement. This statement should provide the following information, where applicable:

- Accession codes, unique identifiers, or web links for publicly available datasets
- A list of figures that have associated raw data
- A description of any restrictions on data availability

All data are available within this study are available from the corresponding author upon reasonable request. Deposited RNA-Seq data, GEO: GSE120079. Source Data represented graphically in the figures are available in "Source Data".

Field-specific reporting

Please select the best fit for your research. If you are not sure, read the appropriate sections before making your selection.

☒ Life sciences ☐ Behavioural & social sciences ☐ Ecological, evolutionary & environmental sciences

For a reference copy of the document with all sections, see nature.com/authors/policies/ReportingSummary-flat.pdf

Life sciences study design

All studies must disclose on these points even when the disclosure is negative.

Sample size	No statistical methods were used to predetermine sample size. How to choose sample size is indicated in corresponding Figure legends.
Data exclusions	No data was excluded from the analysis.
Replication	All the experimental findings were reliably reproduced. The replication is indicated in corresponding Figure legends.
Randomization	The randomization is indicated in corresponding Figure legends.
Blinding	The investigators were not blinded in regard to allocation of samples during experiments and outcome assessment. For RNAseq and Patch-Clamp recordings, the investigators were not blinded.

Reporting for specific materials, systems and methods

Materials & experimental systems

n/a	Involved in the study	
☒	☐	Unique biological materials
☐	☒	Antibodies
☐	☒	Eukaryotic cell lines
☒	☐	Palaeontology
☒	☐	Animals and other organisms
☒	☐	Human research participants

Methods

n/a	Involved in the study	
☒	☐	ChIP-seq
☐	☒	Flow cytometry
☒	☐	MRI-based neuroimaging

Antibodies

Antibodies used	The dilution of antibodies and reagents used in this work are indicated in Supplementary Data 1.
Validation	For each antibody we used, a unique Research Resource Identifier (RRID) is provided for validation information.

Eukaryotic cell lines

Policy information about cell lines

Cell line source(s)	Human embryonic stem cell lines: H9 (NIH approval number NIHhESC-10-0062), H1 (NIHhESC-10-0043); Human pluripotent stem cell lines: iPS (IMR90).
Authentication	H9 (NIH approval number NIHhESC-10-0062), (NIH approval number NIHhESC-10-0043), iPS (IMR90)
Mycoplasma contamination	All cell lines tested negative for mycoplasma contamination.
Commonly misidentified lines (See ICLAC register)	No commonly misidentified cell lines were used

Flow Cytometry

Plots

Confirm that:

☒ The axis labels state the marker and fluorochrome used (e.g. CD4-FITC).

☒ The axis scales are clearly visible. Include numbers along axes only for bottom left plot of group (a 'group' is an analysis of identical markers).

☒ All plots are contour plots with outliers or pseudocolor plots.

☒ A numerical value for number of cells or percentage (with statistics) is provided.

Methodology

Sample preparation	Cells were dissociated with Accutase and further dispersed into single cells. Then washed three times with cell staining buffer (BioLegend, 420201). For hESCs, cells were fixed in 4% (v/v) paraformaldehyde in PBS for 20 minutes. After three times of washing, B2M staining (FITC anti-human β2-microglobulin Antibody, BioLegend, 316304) were performed for 60 min on ice in FACS buffer. For detect and measure median fluorescence intensity (MFI) of AAVS1-PC-mNG-SMASh hESCs or AAVS1-PC-mNG (GGS)-SMASh hESCs, FACS were performed on live cells without fixation.<br>To analyze the cell cycle of a cell population using PI staining, single cells were harvested as described above. Cells were collected in 1.5 mL Eppendorf tubes and then resuspended in 1 mL of chilled 70% ethanol overnight at -20 ºC. The next day, cells were washed and resuspented in 200 µl Muse cycle reagent (Muse cell cycle kit, Millipore, MCH100106) for 30 minutes at RT in the dark. Finally, cell cycle profiles were measured using a BD FACSCalibur flow cytometer.
Instrument	BD FACS AriaIII flow cytometer
Software	FlowJo_V10, ModFitLT V3.2.
Cell population abundance	The respective information is provided in Supplementary Fig. 12.
Gating strategy	The respective information is provided in Supplementary Fig. 12.

☒ Tick this box to confirm that a figure exemplifying the gating strategy is provided in the Supplementary Information.
